# Operationalizing the distribution of oral HIV self-testing kits to men who have sex with men (MSM) in a highly homophobic environment: the Nigerian experience

**DOI:** 10.1186/s12889-021-12378-0

**Published:** 2022-01-06

**Authors:** Adekemi Sekoni, Waimar Tun, Osasuyi Dirisu, Temitope Ladi-Akinyemi, Elizabeth Shoyemi, Sylvia Adebajo, Folasade Ogunsola, Lung Vu

**Affiliations:** 1 Department of Community Health & Primary Care, College of Medicine, University of Lagos, Idi Araba, Lagos, PMB: 12003 Nigeria; 2 HIV and AIDS Program, Population Council, Washington, DC 20008 USA; 3 HIV and AIDS Program, Population Council, Utako, Abuja, 900108 Nigeria; 4 HIV and AIDS Program, Population Council, Yaba, Lagos, 100001 Nigeria

**Keywords:** KOLs, MSM, HIVST, Operationalization

## Abstract

**Background:**

Self-care health interventions are innovative approaches for improving health and achieving the sustainable development goals (SDGs). Men who have sex with men (MSM) have been disproportionately affected by Human Immunodeficiency Virus (HIV). In spite of this, stigma from healthcare workers has reportedly prevented MSM from accessing HIV testing in health facilities. This paper explored the operationalization of using key opinion leaders (KOLs) to distribute HIVST (HIV self-test) kits to MSM. This qualitative survey used a combination of in-depth interviews (IDI) with HIVST users and focus group discussions (FGDs) with KOLs to collect data three months after the distribution of the test kits by the KOLs. Thematic analysis of the data was carried out.

**Result:**

Three themes were generated namely: KOLs serve as a trusted resource to promote and support HIVST for the MSM community; Skills and qualifications required for KOLs to effectively distribute and promote uptake of HIVST; and Effective strategies used to create demand and promote uptake of HIVST.

**Conclusion:**

This study showed the practical steps involved in operationalizing KOL support system distribution of HIVST that positively influenced the testing experience for the participants irrespective of the HIV status and engagement in care. KOLs are a reliable resource to leverage for ensuring that HIV self-test kit is utilized and HIV positive individuals are linked to treatment and care in homophobic environments.

**Supplementary Information:**

The online version contains supplementary material available at 10.1186/s12889-021-12378-0.

## Introduction

In several countries, available evidence suggests a high prevalence of HIV infection among men who have sex with men (13% in Togo; 17% in Nigeria; 22% in Tanzania) [1–3]. In sub-Saharan Africa, this population group is five times more likely to be living with the virus compared to men within the general population [4]. Moreover, this occurs within the context of low utilization of HIV testing services linked to sexual stigma from healthcare workers. Sexual stigma inhibits engagement with HIV testing resulting in high levels of undiagnosed HIV infection. This type of stigma manifests as enacted, felt and internalized stigma [5, 6].

The trend of HIV infection reported by national surveys in Nigeria showed that MSM constitutes the largest population group affected with an increase in prevalence rate from 13.5 to 22.9% over seven years (2007 to 2014) [1, 7]. The conflict between traditional norms, religion, societal expectations versus homosexuality has created a hostile, homophobic, and sometimes dangerous environment for people involved in or suspected of same-sex relationships [8, 9]. The situation was exacerbated by the Same-Sex Marriage (Prohibition) Bill of 2014 which forbids any cohabitation between same-sex sexual partners and bans any public show of same-sex amorous relationship. A prison sentence was clearly defined for offenders and supporters [10, 11]. Evidence exists that before the bill, MSM in Lagos were experiencing stigma, discrimination and social oppression [12]. Since the passing of this law, the social environment in Nigeria became more hostile [13]. The effect of this law spilt over into health services resulting in discrimination by healthcare providers [14] and subsequently a reduction in uptake of HIV-related prevention, treatment and care services in health facilities [15].

The World Health Organization (WHO) recommends HIV self-testing (HIVST) as a tool for improving uptake of HIV testing services and achieving the UNAIDS 90-90-90 target [16]. HIVST as an additional strategy has the potential to increase HIV testing behavior among MSM in Nigeria most especially first-time test takers. This mode of testing makes it easier for MSM who are unwilling or unable to go to the regular testing site to avoid stories/actual experiences of widespread sexual stigma. It is a process whereby an individual collects their blood or oral fluid and performs the test by themselves with the aid of the instructions in the test kit or while receiving help from a trained person [16]. Evidence exists that deployment of HIVST improves uptake of HIV testing among men [17–20] in several SSA countries implementing HIV self-testing including South Africa, Zimbabwe and Botswana [17–19].

In 2017, the Population Council, in collaboration with the University of Lagos, carried out an implementation research project testing the feasibility of a distribution intervention for oral HIVST kits to MSM living in Lagos using key opinion leaders (KOLs) [21]. KOLs are peer educators who are trusted, well-respected MSM, trained to encourage their MSM peers to seek health care, get tested, and access HIV prevention and treatment services. This project was the first implementation science research project to examine a distribution modality for HIVST kits among MSM in Nigeria [22]. In this paper, we describe the operationalization of this strategy based on qualitative interviews with KOLs and MSM who participated in this study.

## Method

### Study design

This qualitative study was conducted as part of a larger study that examined the HIV testing uptake and outcomes of the intervention that took place in 2017. Details of the study and its main findings can be found elsewhere [21, 22]. In this study, focus group discussions with the KOLs who distributed the HIVST kits and semi-structured in-depth interviews (IDIs) with MSM who received the kits were conducted three months after the kits were distributed.

In this study the Key Opinion leaders who are peer educators also served as peer navigators for MSM who tested positive with the HIVST kit.

### Description of HIVST distribution

This study employed and trained twelve KOLs aged 18 years and above to recruit MSM from their personal networks into a baseline cohort. These KOLs were selected from the pool of KOLs trained to provide HIV Testing Services (HTS) by Population Council’s MSM-friendly community-based health center (CHC) in Lagos. These included KOLs who self-identified as MSM or gay, had at least secondary education, possessed good interpersonal skills, and had current information about their MSM community, events, and hotspots (physical or virtual) in Lagos state. The KOLs were diversified in age and professional and education status. KOLs mobilized MSM peers at hotspots considered to be safe, such as football fields, clubs, parties, gyms and cafes. Virtual mobilization was done via mobile applications (primarily WhatsApp). KOLs informed peers about the study and provided them with information or referral cards to either meet at the Population Council’s MSM-friendly community health center (CHC) which offers HIV prevention and treatment services or another convenient and safe space for the study screening, interview, and HIVST kit distribution. To be eligible for the study and receive the HIVST kits, participants had to be biological males aged 17 to 59 years, report anal intercourse with a man in the past 6 months, self-report HIV negative or unknown status, have no recent HIV test (< 3 months), have lived in the city of Lagos for the three months prior to the interview, and plan to stay in Lagos for the next four months.

Eligible participants completed a baseline survey administered by research assistants who are members of staff of the Population Council’s MSM-friendly community health center (CHC), they also conducted interviews at endline and performed follow-up calls. Upon completion of the interview, participants received a package that contained: (1) two HIVST kits (OraQuick Rapid HIV ½ Antibody, OraSure Technologies), a rapid oral fluid-based test kit, (2) a referral voucher to access free HIV testing at the CHC, (3) information about the hotline, and (4) HIV prevention and HIVST information leaflet, condoms and lubricants. Furthermore, the KOLs shared with the participants a video in English that demonstrates how to correctly use the HIVST kit.

Due to mental health concerns in case of a reactive test result, a phone hotline was set up for four months and managed by a certified HTS counsellor to provide support for usage and facilitate referrals for HIV treatment and other support services for those testing reactive. In addition, the HTS counsellor followed up with participants by calling the participants on days 5, 30 and 80 after participants received the HIVST kits.

### Data collection procedures

A follow-up quantitative survey (Endline) was conducted after three months with the baseline cohort to determine whether participants had used the HIVST kits and their experience with them, their self-reported test result, potential harms, confirmatory testing (if reactive), linkage to ART, and willingness to pay for HIVST. From this pool of participants who completed the 3-month survey, a purposive sample of 20 participants was selected to participate in IDIs. Participants were diversified by age, education, and self-identified sexual orientation. An effort was made to select participants who used the kit as well as those who did not use the test kit; however, the majority had used the kit (97.7%) and thus we were unable to interview participants who had not used the test kit. The IDIs explored MSM’s self-testing experiences, understanding of HIVST instructions, reasons for use/nonuse of the test kits and linkage to post-test counselling and HIV care, if positive.

Ten out of the twelve KOLs employed in the intervention participated in two FGDs moderated by a trained research assistant. The FGDs were conducted to assess the operational aspects and challenges to HIVST kit distribution. Data collection took place between September and November 2017 at the CHC.

### Data analysis

Data from the IDIs and FGDs were recorded in real time, transcribed and translated without any personal identifiers. The procedure for data analysis was the same for the FGDs and the IDIs even though the analysis was conducted separately. Thematic analysis of data was used. The transcripts were divided into five batches and shared among five researchers for initial independent reading and manual coding. This was followed by a meeting to discuss the initial codes and the first set of a codebook. At this meeting, the five coders went through all the transcripts together generating a refined codebook and a thematic table. The third and final round of coding was carried out using Nvivo 11 during which overlapping themes were integrated, and the codebook and the thematic table were also condensed.

### Ethical considerations

Ethical approval for the study was obtained from the Population Council Institutional Review Board (New York) and the College of Medicine of the University of Lagos Health Research and Ethics Committee.

## Results

The following socio-demographic characteristics of KOLs were observed. 40% were 24 years of age or older while 60% were younger; 30% had some or had completed secondary schooling while 70% had postsecondary school education; 80% were employed, 10% was a student and 10% was unemployed.

The following socio-demographic characteristics of IDI participants were observed: 45% were older than 24 years old; 30% had some or had completed secondary schooling while 70% had postsecondary school education; all were single and not living with a sex partner; 65% identified as bisexual and 35% as gay men.

Findings are discussed through three thematic areas:

### Theme 1 - KOLs serve as a trusted resource to promote and support HIVST for the MSM community

*I had a lot of feedback when I gave them the kit and they get to use it. They called me, they thanked me, and even those ones who ended up finding out that they were positive, they were happy. (KOL, 22 years old, Tertiary education)*

*For me, throughout this study, the thing I love is that most of the people I recruited accepted it and I followed them up. They open up to me which made me so happy. The five people that I recruited that were reactive had not accessed the HIV testing services before. I invited them they accepted, they know their status and their success stories is that they accepted the result and I linked them to care. They are now doing fine. (KOL, 25 years, Post-secondary diploma)*

*Em ... distributing the kit has not been easy and, also, in a community like ours whereby there are rules that restrict MSMs …. they will like, tell you that 'are you the only one coming', if you say 'no, I’m bringing somebody they will ask you another question is the person coming with you a community member [MSM] ?', that is one clause already and the next thing is 'will I be asked questions ?' and if you say ah! you will just be asked a few questions, and if he says will he ask questions about my sexuality or my sexual orientation? and you are like 'yes', it’s another clause already, so it restricts them. (KOL, 20 years old, Post-secondary diploma)*

In recruiting participants, KOLs reported that most of the eligible individuals they approached agreed to enroll in the study. Overall the KOLs ranked the HIVST intervention very successful and they were happy with the outcome and the benefit to the MSM community in Lagos state. Establishing trust was a key determinant of participant recruitment, some KOLs reported that the involvement of non-MSM individuals (members of staff of the Population Council’s MSM-friendly community health center (CHC) who conducted interviews at baseline and endline and counsellors who conducted the follow-up calls) caused anxiety, suspicion and reluctance to volunteer information during the enrollment interviews. To overcome this threat, the KOLs engaged in a continuous exercise of assuring participants of their safety at the recruitment site and confidentiality of the information they provided leading to a smooth engagement and willingness to respond to the follow-up calls from the counselors.

*I just spoke with the KOL… [name of KOL] was the one who referred me to the study, I just felt it was not necessary to call the hotline. (HIVST kit user, 24 years old, Tertiary education)*

*KOL distributing the kit is ideal, as they are giving you the test kit they are able to recommend you to a certain health center, you have somewhere to go to, not that you are doing it and then you are just stranded, not knowing where to go to because some hospitals don't actually know what to do….. (HIVST kit user, 20 years old, Tertiary education)*

HIVST kit users reported that they saw KOLs as a safe source of support for them and that they would prefer to reach out to a known and trusted person such as a KOL rather than call an anonymous hotline for support. Many of the MSM participants indicated that their first line of action if they encountered challenges while testing or immediately after testing would be to call the KOL as opposed to any hotline. Access to the same KOL who gave them the kit made participants feel more comfortable. By maintaining phone contact, visiting peers at home, talking to them and staying in touch, the KOLs were a constant source of emotional and psychological support to HIVST kit users. At the same time, this interpersonal communication was an avenue for HIV-related education, pretest counseling, ongoing counseling and post-test counseling. This support is crucial during the follow-up stage of the study and critical to the quality of person-centered care provided by the KOLs. The quality of the follow-up, therefore, impacted the MSM participant’s mental health and ability to manage a reactive test result. Some self-testers were therefore of the opinion that KOLs should be at the forefront of HIVST kit distribution within the MSM community. According to the KOLs, the personal conviction of duty and commitment to their peers was the driving force for the successful implementation of this intervention.

*……. because people don't want to meet up again due to what happened so it took extra effort from the KOLs to reach MSM. When we started the endline, the Research Assistants were supposed to interact directly with the participants, they were to call and book them for the interview but then she recorded that it wasn't as easy because participants were not responding but the moment she brought in the KOLs as intermediaries, everybody started coming in and that's because we have been able to establish trust...so I think that's the effect of the KOLs and trust... (KOL, 23 years old Undergraduate)*

*…at this point there's nothing like you gathering with five MSM because the government is on it, everybody is on it, so you gathering up to five MSM is a high risk …me, I’m scared… me, I don’t want to go to prison now, my mama no know say I be homosexual, you don't need to gather five people or six people because now everywhere dey hot, everywhere is hot now. (KOL, 24 years, Completed secondary school)*

During the study period, the social environment in Nigeria was particularly challenging for MSM due to security-related concerns. Some members of the broader MSM community (not study participants) were arrested at a hotel during a night party, followed by detention in a police station. They were subsequently arraigned for prosecution in a magistrate court in Lagos state. The forty-two detainees had their pictures on social media, newspapers and TV. They were also tagged as HIV-positive individuals resulting in double stigma for the MSM individuals and their families. This was a great source of emotional and psychological distress within the MSM community, especially in Lagos state. Subsequently, to avoid attracting unnecessary attention, community members avoided gathering in public spaces. The recruitment of participants, therefore, was challenged by this environment and hence KOLs focused on one-on-one interactions and kept group gatherings to a minimum. KOLs used their best judgment to identify various venues such as eateries, restaurants, gyms, cinemas, shopping malls considered safe to meet with potential participants.

*A few of them I have been searching for them, I have not been able to reach out to them because maybe they have misplaced their phones. (KOL, 20 years old, Post-secondary diploma)*

In spite of these challenges, loss to follow-up in-between the period of HIVST distribution and the end of the three months follow-up was reported by some of the KOLs. This was uncommon and mainly due to the inability of the KOL to maintain contact with the participants after several attempts using all possible means. During the study period, some MSM relocated to the outskirts of the state while a few relocated to other states for security reasons. The social environment was described by KOLs as “hot” for MSM (i.e., the potential for harassment and arrest for those believed to be engaging in homosexual behavior) necessitating flight to escape suspicion and possible arrest. This was responsible for some cases of loss to follow-up while a few were due to some participants losing their phones and Sim cards.

### Theme 2 – Skills and qualifications required for KOLs to effectively distribute and promote uptake of HIVST kits

*…… one thing I really liked about the training we had before the baseline, we were trained to answer different kinds of questions about HIVST and that helped greatly. Now that you are negative, these are the things to do to continue staying negative, this, if you are reactive [positive], this and this is what to do… (KOL, 23 years old, Undergraduate)*

*…… giving the correct information of what the self-test kit is all about and giving them proper information about the HIV test and HIV in general, and if we have to find out that one of them is positive and they do not want to use this facility, we should make out time to follow them …… they should create confidentiality between them and their client to link them to confirmatory test and post-test. (KOL, 25 years old, Tertiary education)*

KOLs spoke about the importance of intensive training on HIVST given that it was a new concept. Such training should cover other HIV related topics, especially HIV treatment and referrals to treatment facilities so that they will adequately handle the issues that might come up during their conversations with community members. Training of the KOLs on HIVST in this feasibility study built capacity among the KOLs which subsequently had a cascading effect within the MSM community. This allowed for continuous access to reliable sources of information and HIV services for the MSM community.

*…. and then I had to be on the phone, are you ready, let's do this and then the whole twenty minutes of waiting for the result, we had to keep talking and when the results came out and they were fine …. (KOL, 23 years, Postgraduate student)*

This HIVST kit distribution intervention highlighted the social and interpersonal skills needed by KOLs to be effective. The ability of KOLs to manage individuals irrespective of their personality was key to its successful implementation. The intervention required a great deal of support from the KOLs to potential HIVST kit users after the kits were distributed. KOLs reported that the support they provided during house calls and phone calls included: encouraging individuals who claimed they were scared to use the kit, clarifying HIVST kit instructions for users on request, chatting on the phone with individual’s who were extremely anxious during the 20-min waiting time required to read the test result, physically assisting peers who specifically requested for their presence during the testing, referring peers for confirmatory test, escorting individuals to the Population Council’s CHC for a confirmatory testing, and liaising with healthcare providers at other referral sites. KOLs, for the most part, encountered peers who were interested in HIVST and welcomed and requested their support; however, they also reported that they sometimes encountered peers who were rude and unfriendly and peers who warned them to desist from calling their phone lines.

*My experience with the whole DIY study was that for the baseline, there was a little bit of resistance because this is something new, it's expected, people ask questions what is going on here?, and so it was the major job of the KOLs to enlighten these people that okay this is what we are doing, this is why we are doing this and this is what this whole process is about and you discover that at the endline, the participants were the ones reaching out, let's do this test because now they know, now they've used it, now they've seen how good this is, so the KOLs were not doing so much in the endline compared to the baseline (KOL, 23 years old, Postgraduate)*

Patience and persistence was a valuable asset employed by the KOLs in this study. Most of the KOLs reported ease in promoting HIVST, it took some time to build up the momentum for interest in the MSM community. Some of the KOLs considered the early period of the study quite taxing to generate interest. They reported that in the beginning, some of their peers within the MSM community were curious and asked a lot of questions during the sensitization sessions while some were cautious and indicated that they would rather not be tested for HIV. The KOLs considered this to be a normal part of the introduction process of any new intervention. However, after a few months of continued effort, the demand creation had been established within the community, and peers started calling the KOLs and visiting the CHC to request for the HIVST kits for personal use; partners; friends and family.

*……..another thing that is a hindrance to the distribution of the kit is that you giving the kit out to them looks as if you are suspecting them already, they believe that my status is nobody's business, so why trying to bug into my privacy, it's later when we let them know that you are free to use it, you are also free not to use it, that is when they will say ok, ok, ok. (KOL, 20 years old, Post-secondary diploma)*

KOLs were a critical and essential resource in the mobilization and distribution process of this HIVST study named “do it yourself study (DIY)”. Although the KOLs have developed trust with their peers within the community, some reported that during the recruitment process, they had cause to approach individuals who are known to always be on edge and suspicious about anything linked to HIV with caution. Introducing the study to such individuals was considered to be a delicate issue requiring tact and caution mainly because such individuals will interpret the invitation to participate in the study wrongly and become upset. They presume that they are being judged and suspected to be HIV positive hence the invitation to participate in the study. According to the KOLs, they had to be cautious and tactful in those instances. They were able to recruit individuals in this group because they informed them that if they enroll in the study and received the kits, they will exercise their free will in deciding if they want to use the kit or not as well as the time they want to use it. Giving the study participants a choice was especially important because HIV testing is voluntary and the study was interested in knowing the proportion of the study subjects that will not use the HIVST kit and the factors responsible.

### Theme 3 - Effective strategies used to create demand and promote uptake of HIVST

*…I was about to talk about the video you know, if* I *am to send the video to my social media, Facebook and WhatsApp group? I created a group, I have some few MSM, most of them watched the video and they were like we want self-test, that they will like to you know, do the test... the video was a success. (KOL, 20 years old, Diploma)*

*And it was as easy as I saw the video of how to use it and I tested negative with it… at first when I wanted to use it, I thought it would be very difficult, I watched the video then I read the instructions and I just did as simple as that, so no one would find it difficult to use because it's very straight to the point.... (HIVST kit user, 19 years old, Completed secondary school)*

The use of the HIVST demonstration video sent to the participants by the KOLs was considered a selling point by both KOLs and MSM HIVST kit users. The video was not bulky and easily sent to networks and individuals using a mobile phone. The live images allowed easy comprehension of the HIVST procedure by individuals with a low literacy level. The video was sent at varying periods during the intervention phase depending on need and request. Its use extended beyond the enrolled participants to serve as a teaching aid for KOLs in HIVST awareness creation within the MSM community.

*….. Like me, the first day I started writing something on my Facebook, about HIV prevention, people said ah! ah! why are you writing, sending like bulk SMS to people about HIV, I want to get something from them. So, they now started chatting me up privately, that you are writing so so so!!! I like what you are writing, how can I access this thing?… (KOL,* 25 years old, Tertiary education)

*….social media was a huge boost...I don't know how to put it. Social media helped me and my network. By the time I get to tell a friend and then my friend tells his friend...the main thing is while telling the friend or anybody, I give them the eligibility criteria, and it made it easier for me to recruit. So, at the end of the day when I invite one friend for the study, he comes with four other people knowing the fact that he has let them know about the eligibility criteria. It was then left for me to do my own screening and then they are all there for me. (KOL, 22 years old, Tertiary education)*

The KOLs used social media (Facebook/SMS and WhatsApp etc) to initiate conversations around HIVST within the MSM community, thereby increasing the reach and coverage of information about HIVST. Apart from social media, information was sent to contacts via bulk SMS which is a cost-efficient method for reaching individuals who do not have internet access.

*What I really liked about the study was the fact that each client’s wish was respected and their feedback counts. What do I mean by that, for example; a client comes in and he went through the baseline and was given the kit and he decides not to use it. It still counts because we get to ask them, why didn't you use the kit? you understand what I am talking about, I like the fact that every client feedback counted, and their wish was respected. So, it was a YES for me. (KOL, 22 years old, Tertiary education)*

*…….the process of collecting the HIVST kit for me was okay, it wasn't stressful, at least I was in school and the KOL actually brought it down to my school, so it was, it was comfortable for me. (HIVST kit user, 27 years old, undergraduate)*

During recruitment, these platforms helped greatly allowing KOLs to tap into individuals with large networks once the KOLs network gets depleted thereby recruiting beyond their immediate networks ultimately generating demand for HIV testing within the MSM community in Lagos state. KOLs routine activities within the community including free condom and lubricant distribution were leveraged upon to disseminate information about the availability of HIVST, and the study. Routine home visits provided additional ample opportunities for HIVST education and recruitment of willing peers.

The flexibility of having multiple recruitment sites enabled MSM who will rather not identify with the gathering of MSM individuals or with the CHC to participate. The reluctance of some eligible participants to enroll at the CHC stems from the perception that the CHC only provides services to HIV positive people while for some people, their friends/acquaintances work at the CHC and they will rather keep their affairs private. Such individuals were directed to alternative venues by the KOLs for enrollment and in some instances, the kits were hand-delivered to the participants by KOLs because of logistics reasons. Population Council (PC) linkage with public health facilities was leveraged on as such individuals were encouraged and assisted by the KOLs to visit the nearest PC trained public health facility for confirmation and linkage to care if necessary. The KOLs were invaluable in the referral and linkage component of the study. They were also responsible for the sensitization and preparedness of staff in the facilities mentioned above to receive MSM clients.

The language barrier was mentioned as a hindrance to recruitment by some of the KOLs. Not only did it delay achieving individual targets for the KOL, it also limited the diversity of participants they were able to enroll. Of note was the challenge with recruiting individuals with a low literacy level. Some KOLs overcame this barrier by asking their peers for assistance during screening or by simply inviting the individual to the recruitment center to enable the appropriate research assistant to screen for eligibility. Some KOLs opined that recruitment would have been smoother and would probably have yielded a more diversified sample for the study if they had been collectively assigned group target rather than each given a target to recruit. This method will enable them to leverage each other’s strengths while minimizing individual weaknesses for the recruitment.

To encourage the participation of individuals working in the formal sector, the study team worked on Saturdays. This group of individuals were constrained because of their tight schedule during office hours and at the same time, the study team could not go to their workplace to recruit them.

*We didn't emphasize on older people, how you can walk up to elderly people and tell them about the HIV self-test kit. We need more training when it has to do with approaching elderly people. There is a difference between high class and elderly people, people in their 40’s, 50’s, 60's the way I approach them wouldn't be the same way I will approach my friends in their 20's. (KOL, 20 years old, Post-secondary Diploma)*

*….I don't have the power to recruit those elderly ones because I mingle with my class you cannot see me playing with an elderly person, I mingle with my age, I mingle with adolescents. Maybe we have those that are supposed to recruit adolescence and the KOLs that will recruit the older ones so it will be balanced. (KOL, 25 years old, Post-secondary diploma)*

Especially challenging in this study was the recruitment of individuals referred to as older men (40 years and older) and men of higher socioeconomic status, many of whom live on the Lagos Island. In hindsight, some KOLs observed that the pre-intervention training should have included how to specifically recruit these subgroups of men. Other suggestions for improvement from the KOLs include using KOLs from all the identified groups for recruitment. In this study, this challenge was partly solved by identifying and using links to older community members that offer services to this category of men.

## Discussion

This paper presents the issues arising during an implementation study that operationalized KOL mobilization, distribution, follow up, referral and linkage to care for HIVST kits among MSM in a criminalizing environment. The findings from the study are unique and contribute to the literature geared towards ending the HIV epidemic. Specifically, it highlights how the use of KOLs/peer educators can be operationalized to improve the reach of HIV testing which is extremely useful for programs. Other papers on this topic have shown that peer educators are effective but unlike this paper do not offer an in-depth qualitative examination of how KOLs should be used effectively [23–25]. This paper provides very highly practical operational approaches and learnings for programs. The importance of establishing trust with MSM communities in health-related interventions in a highly homophobic environment like Nigeria was clearly demonstrated in this study. Leveraging on the social networks of KOLs ensured recruitment, engagement and retention of participants within a short period of time.

This study effectively demonstrated the importance of reliable and trusted follow-up support. One of the biggest concern raised about HIVST is that people will not receive appropriate counseling, and also not be linked to treatment and care [26–28]. The support received by MSM from KOLs during the testing stage is critical in linking people who test positive to services for confirmation of HIV status, treatment and care. This will ultimately contribute to the end of the HIV epidemic by 2030. At the same time, the KOL support system in this study positively influenced the testing experience for the participants irrespective of the HIV status thereby contributing to the acceptability of this testing model and demand creation within a short period of time. Moreover, the fact that the participants preferred to call the KOLs rather than use the hotline and that loss to follow was rare among the participants further underlines the importance of the role that KOLs undertake in delivering the HIVST intervention.

Studies have shown that HIV testing and a positive test result can be a traumatic experience with mental health consequences. For the key populations that are at higher risk of HIV, the ability of individuals to cope with a positive test result and get enrolled in treatment is therefore paramount. Also, evidence exists that as a result of discrimination and stigmatization, sexual orientation and gender identity is associated with some stress related mental health disorders including anxiety and depression [29–33]. An MSM who tests positive is therefore at the intersection of two marginalized identities and can therefore be at higher risk of mental health disorders. The huge role KOLs played in enhancing the psychological and emotional health of our study participants shows that they are an important resource in promoting and maintaining the mental health of HIV positive and negative men thereby filling the huge gap created by an inadequate health workforce. As a result of evidence generated by this feasibility study, the 2020 national HIV treatment guideline in Nigeria [34] recommends amongst others, HIVST for key population (MSM, female sex workers, people who inject drugs and transgender women) the current program for delivery includes training of KOLs on the use of HIVST and deployment of the trained KOLs within the MSM population to distribute HIVST and other relevant information to their peers via one on one discussions or dedicated social media platforms. Access is further enhanced by community based organizations, community pharmacists and key population clinics who provides HIVST kits via online and physical distribution models.

## Conclusion

Within the context of the ongoing COVID-19 pandemic and public health efforts to prevent the spread of the virus such as lockdown and social distancing, the role of the KOLs in the distribution of HIVST kits to their peers is important for ensuring that people who need to get tested do so and are supported throughout the continuum of treatment and support. KOLs are therefore a powerful resource for minimizing disruption to HIV prevention and care services.

This study was carried out in a cosmopolitan city among a relatively educated population, and thus, the findings may not apply to a less educated population or other parts of the country. Additionally, since this was a small pilot study, we did not have a large enough sample size to be able to stratify our findings by age, professional status, sexual self-identity and other salient characteristics. Also, we had only a few participants who did not use the HIVST kits, and we were not able to reach and interview those people so we were not able to capture their perspectives, which could have been useful. Further research studies should examine how the use of KOLs may work differently for different segments of the MSM community. Despite these limitations, this study has detailed how KOLs can be used effectively to reach MSM with HIVST. Further, it sheds light on how new intervention approaches may be introduced into this community within the context of a highly homophobic and dangerous environment.

## Additional File

Additional File (DOCX 112 kb)

## Data Availability

All data analysed during this study are included in this published article [and its supplementary information files].
