# Supplementary material for: Operationalizing the distribution of oral HIV self-testing kits to men who have sex with men (MSM) in a highly homophobic environment: the Nigerian experience
Source: BMC Public Health. 2022 Jan 6;22:33. doi: 10.1186/s12889-021-12378-0 (PMC8734127; doi:10.1186/s12889-021-12378-0)
Supplement: Supplementary file 1 — Additional File (DOCX 112 kb) [file 12889_2021_12378_MOESM1_ESM.docx]

**MSM HIVST**

# ACCESSIBILITY OF HIVST

This describes the ease of access (physical and financial) preferences of channels for key population to obtain the HIVST kit. This also includes barriers and challenges.

## Affordability

### Fee or free

*JENNIFER:... if you are saying they should sell it, ok for instance, if you say you want to sell this for one thousand naira, when majority of MSM are thinking that they should come around and collect transport after you test them, you now want to sell, they will just tell you that hold your test, we don't want it...*

***KOL FGD_01***

*JENNIFER: Yes! it should just be free of charge because they have been doing free test before and, now if you go to party to go and share this test kit in the party and you are giving someone and say you will pay one thousand naira to get this, the person will just tell you fine, they might like it, they might want it, they might want to use it but they might not have the money to buy it*

***KOL FGD_01***

*TEWO: For sales of the kit, I think it's in two ways, yea, like he said, not everybody is equal but it gives ownership to you for you take money and buy. A student can afford to buy recharge card into their phone, a person who is MSM can always network because they always WhatsApp, we buy data of one thousand five, two thousand naira, which lasts us for a month and test kit is just for once in three months, for heaven's sake now, come on, it is...is cost eh...people pay money to do all these. What I’m only saying is if like I said earlier on when I first started, I said accessible and affordable, may be you guys did not listen properly, I know why I said that accessible in the market for community members and affordable, so that even if it's twenty naira, let it be twenty naira, if it's ten naira, let it be ten naira, so that even ‘A’ who is rich can afford to buy for hundreds of MSMs, but now let's look at those young adolescents who are in schools, whose trouble, who don't have homes, who barely have three square meals in a day, what are we now saying to them, that's why I said it's in two ways*

***KOL FGD_01***

*DOYIN: And that's because now they've used it, now they are in the know and so now they want more, so when you ask them, are you willing to pay, most would say yes because this kit is very easy to use. The moment you leave it free, you get, you can get ten from ten different places if it is free it is very easy to abuse but I feel we should put a little token*

***KOL*** ***FGD_01***

*…make the first one free, but sell the second one*

***MSM_IDI_02***

*I: ﻿Ok, do you mind buying it?*

*﻿R5: ﻿Yea, I don't mind, it's something somebody surely invented it, so ﻿we need to commend his good work, we should buy.*

***MSM_IDI_05***

*R6: ...if it could be sold at an extremely subsidized price, that would be fantastic but if not, if it could be sold at a reasonable price...everybody can afford, then yes I can love that as well, I will actually prefer that*

***MSM_IDI_06***

*R8: Yea, for sale because if it's free it'll be abused*

***MSM_IDI_08***

*It should be free because people run away from such things, I don't know, it should be free.*

***MSM_IDI_13***

*I: Ok! How would you like to obtain this self-test kit, is it through a KOL or coming directly to the community health center or would you like to purchase it, or is there any option?*

*R 15: Purchasing it! No!*

***MSM_IDI_14***

*P.T 1: For me to avoid any kind of abuse in making use of the kit I will suggest (for those testing) if it is for the first time, it should be given free, but at some certain point, I think it should be sold because for it being sold, people won't abuse it because they feel it's their money, they wouldn't just want to waste their money. They will want to make use of it properly so that they will get their test result*

***MSM_IDI_21***

*I: How would you prefer to get the kit, would you prefer to get it through the key opinion leaders again or would you prefer to get it from the CHC here or would you like to buy it?*

*M2: Hmmm! buy! I wouldn't want to buy*

***MSM_IDI_22***

*R8: I doubt, it's still something new, may be later, may be in the next five or ten years it can be free but for now it should still be for sale*

***MSM_IDI_08***

### Possible prices

*TEWO: If the test kit should go for a particular rate, I think for me my own pocket, between five hundred...two hundred to five hundred naira for me, so that any person can afford it…*

***KOL FGD_01***

*DOYIN: Now this token should not be so much in the sense that it boost the resistance. I still agree with the five hundred naira because if you have five hundred naira, I think anybody, if at all you have to save up, it's worth it because it is you knowing your status, you save up for the five hundred naira and you get your kit and you use your kit but the moment it is free, you discover it is very easy to use, so you can use five in a day and that's basically wasting resources, but if you buy five, that's two thousand five, you would never use five in a day*

***KOL FGD_01***

*SEYI: Like may be three hundred*

***KOL FGD_01***

*OLUMIDE: So why now will they test themselves and be positive, do you get, so even if we are going to sell it, it should be a token, that they will be like ok o at least, it's kind of cheap, so I’m saying hundred naira*

***KOL FGD_01***

*R 2: Ok, let me say, five hundred or one thousand naira, so it will be easy for them to buy*

***MSM_IDI_02***

*...maximum of a thousand, I think so, is something someone can get cosI believe someone can get a thousand naira to pick it up.*

***MSM_IDI_03***

*R5: Ok, the self-test kit, hmmm, a thousand naira… Minimum… it could be above one thousand*

***MSM_IDI_05***

*R5: ...we are living in a world that nothing is generally acceptable, what can be rich to some people can be poor to some people, what can be poor to some people can be rich, so I'll just on a scale on just Nigerian economic scale, may be five hundred*

***MSM_IDI_05***

*R6: I think for something like this, I would consider...Two thousand, five hundred…*

***MSM_IDI_06***

*R8: Ok, that everybody can afford, five hundred is okay*

***MSM_IDI_08***

*R 11: May be like, two thousand, three thousand naira*

***MSM_IDI_10***

*R12:...because people are already making excuses that it's too painful, I don't want another excuse like it's too expensive, so maybe it can sale for N500 or N250, you understand!*

***MSM_IDI_11***

*R 13: N1000 should be fine, N500 will be fine. The reason why I am thinking of because if I have to get it for a price, there is no point for me transporting to a centre then I will still get it for a price, it's just the same thing I get it from a pharmacy close to me, and then at that note the price too will limit any form of over usage, abuse too…*

***MSM_IDI_12***

*At the first time, the guy that actually interviewed me, he asked me about this and I said it has to be very cheap so people can afford this thing, I think N200 is ok (two hundred naira) what people can actually afford.*

***MSM_IDI_16***

*R10: if it is to be sold, it should be sold for one thousand naira, because giving it out free, people will just take it and they will misuse it, so it should be sold.*

***MSM_IDI_20***

*M2: ...I can say between five hundred (#500), six hundred (#600)*

***MSM_IDI_22***

## Availability of test kit

*Availability, like some people when they get used to this kind of kit, they will hardly go for test like they will tell you that if they go for test they will prick them and all that, once the kit is available, so many people will get used to it and everybody will like it.*

***KOL FGD_01***

*JENNIFER: And I said this thing before to Aunty Waimar, see this thing if them no go continue am make them no carry am come, this thing they must continue o because people are asking, they want to kill me, they want self-test kit, they want oral self-test kit. Abeg they should bring it back, so people can be using it, so we no go lost all these three hundred people like that, because e be like say this three hundred people among them now we have some people that say they don't want pin (needle prick), they want to see their blood, so that's why we still need it back, among them might say they don't want to come to Pop Council, I can remember some people that their phone is switched off, you just see them like magic in the clinic, we have some people they will tell you that “wo! I’m okay, mi o wa mo, mo ni'se” [Yoruba meaning, I’m not coming again, I have work] but we going to their house and be sharing this thing for them, that we still dey and we still have interest in you, so they should just continue.*

***KOL FGD_01***

*I: You bought this self-test kit (he asked with surprise)*

*R 2: Yes, I bought another one for myself… At Ikorodu pharmacy*

***MSM_IDI_02***

*I: do you think this oral self-test kit should be made available widely?*

*R5: Yes! It should*

***MSM_IDI_05***

*R6:...I don't know if it could be made available to stores for purchase, that would be fantastic, not only would it be easily gotten that's if it's cheap though but it can always be, it can always be gotten more because since they are making money off it they can always pick more but as opposed if I'm receiving it from here and they give it to me free, you can always run out, so at a time when I feel like I need it and they don't have it, I will let down if there's especially if no other option to get one*

***MSM_IDI_06***

*availability like some people when they get used to may be this kind of kit, they will hardly go for test like they will tell you that if they go for test they will prick them and all that, once the kit is available, so many people will get used to it and everybody will like it.*

***KOL FGD_01***

### Future of continuous supply

*those people I recruited when they started showing their friends they started calling me that they need it, I told them it's not yet fully here in Nigeria but let's pray that this type of kit comes in here in Nigeria because we notice that most of our community members they don't like the normal one, they love this oral self-test.*

***KOL FGD_02***

## Barriers to accessibility

### Loss of contact

*I think two or thereabout didn't come in for the self-test because I lost their contact during that period in time, I think they lost their contact also during that period, I have not been able to reach out to them*

***KOL FGD_02***

*some of them I have been able to search for them, I have not been able to reach out to them because of maybe they have misplaced there phones.*

***KOL FGD_02***

### Distance

*…another thing that he said that really triggered something interesting is distance*

***KOL FGD_01***

### Stigma, Trust and Privacy issues

*DOUBLE D: ...distributing the kit have not been easy and also in a community like ours whereby there are rules that restrict MSMs, so, even like meeting a fellow MSM to give him or her the kit, it limit us from giving them the kit because the issue of confidentiality is very important and most times they will like tell you that 'are you the only one coming', if you say 'no, I’m bringing somebody that will ask you question and the person is not a community member', that is one clause already and the next thing is 'will I be asked questions' and if you say ah! you will just be asked few questions, and if he say will he ask questions about my sexuality or my sexual orientation and you like 'yes', is another clause already, so it restrict them. Another reason again that I can say is a hindrance to the distribution of the kit, you giving the kit out it look as if you suspecting them already, they believe that...whatever my status is nobody's business, so why trying to bug into my privacy, it's later when we let them know that you are free to use it, you are also free not to use it, that is when they will like ok, ok, ok.*

***KOL FGD_01***

*TEWO: Because not everyone will want to even open up if they don't have a strong connection with you*

***KOL FGD_01***

*people feel ok come in to a particular facility and everybody here knows what I’m coming to do here may be because everybody has the idea, the mindset that ok people who are HIV positive apparently comes here, so the fear of them coming to a particular facility, it's another issue…*

***KOL FGD_01***

## KOLs

### Experience with recruitment/mobilization

*DOUBLE D: ...I was able to convince some of them that I’m not trying to intrude in your privacy and whether you use the kit or not... it's not important, just accept the kit, I’m not asking you to tell me your status after using the kit, anything that...it's your result, is your personal issues, there are hotlines there that you can call and if you are not free coming to any TB, any of the MSM centre, you are free to go to a general population, a community health centre to access care, I even went as far as some of my clients that are living close to Igando, I had to go to Igando to drop that our short card talking about the CHC, to the heart to heart centre that in case you see anyone coming with this, just know that this is what is going on and they were able to...ok, no problem*

***KOL FGD_01***

*JENNIFER: ...people say yes to it because they believe that is the easy thing for them, them pricking themselves, a lot of people don't like pricking and all of that, so they say yes to this…*

***KOL FGD_01***

*TEWO: ...the first part of it is accessibility and availability of it and the willingness of respondents to apparently use it freely and let me take the key points; the first time we met we discussed on the interviewer interviewing them and gave them the pack and what I’m trying to point out is, it is a brand that will sell in the community because it easy! you can even check it yourself, it's available at every time you need it, not as in sell... the mindset that ok people who are HIV positive apparently comes here, so the fear of them coming to a particular facility, it's another issue but you giving them that thing, the test kit, which is oral, for them to do it themselves and know their status themselves, I think they will seek for another informed... knowledge or comprehensive knowledge which they can acquire from a phone line, I think the phone line was being given to them to call and by that time they can fix extra days, extra hours, may be like ok after work for the working class persons, ok if the facility close by four, then make it, you can come on Saturdays, find time and it will really help a lot of busy persons, people who have their shops, who are self-employed, and this is the platform for them to really access health services, that's what I think.*

***KOL FGD_01***

*DOYIN: ...my experience with the whole DIY study was, for the first baseline, you would discover there was a little bit of resistance because this is something new, it's expected, people ask questions and what is going here, and so it was the major job of the KOLs to enlighten these people that okay this is what we are doing, this is why we are doing this and this is what this whole process is about and so you discover that the endline, the participants were the ones reaching out, let's do this… the participants were the ones because now they know, now they've used, now they've seen how good this is, so the KOLs were not doing so much in the endline compared to the baseline, the baseline we were the ones telling them, come, come and see how this works, let's see if this is feasible in Nigeria…*

***KOL FGD_01***

*SEYI: …my little experience I had from the baseline… we were able to pass the information correctly to the extent that they were even the one calling for the endline*

***KOL FGD_01***

*OLUMIDE: ...it went well, so the thing is that selling the kit is not something we should go into because some people don't care about their HIV status, I know about that, some people don't want to know, they will just be like shey he is living fine lati'jo yi…*

***KOL FGD_01***

*JENNIFER: Ok, things I like about the project, if I remember I have these set of friends that I have been begging them to come around for HIV test, they have not been tested before, they don't even know anything about HIV and they don't want to know anything about it, so me discussing oral self-test with them, telling them how it works and all of that, they like 'oh really!' ok they will like to do it, they want to do it, so it make us...it make us to bring back all these denials that saying that I don't want to do test, I don't want to know my status, it make us to bring them back, I can remember that the first week we started this project we have a lot of people, a lot of new people, even though Charles upstairs asked me question that are you sure this people are not just, did not just start MSM yesterday? I was like they are MSM, ask them question, Elizabeth started asking people question and Elizabeth was like are you serious! So these people exist, so they are here and don't know anything about HIV at all, I’m very happy, even after, the endline you have a lot of people coming in and saying that...new people o, I’ve not even seen them before, talking that they want this test kit, I was like where are these guys before, where una dey?*

***KOL FGD_01***

*DOYIN: One thing I like about this survey was it kind, it exposed me to different kind of people, you know, I always had the impression people are ready to contribute to their community and all that but with this survey it made me realize some people are actually holding back, they don't want to have anything to do with any of these things because you meet some people and you tell them we are going to be giving you transportation reimbursement and they are like no, why, I don't need this, I just want to contribute my quota to the community and I meet some people who come majorly for the money than for any other thing, so it exposed me, it enlightened me that ok, we need to understand that there are different classes and groups of people and this product has to serve all round, all groups, you get, it's like condoms...some people will rather pay for condoms where they give condoms free, so I think if we okay well what I like is the survey helped me understand this and then if there's anything I do not like is the fact that some people end up positive and the whole process of denial and everything does not allow them to access care, so I am here thinking how do I make this person access care, how do I even convince him to come around because he has found out the result by himself, he has been confident enough to tell me and yet he doesn't want to access care because he is in denial and he is, so that's one fear I have, how do we make sure people who end up reactive access care*

***KOL FGD_01***

*TEWO: ...for me I would say I love the willingness of respondents to participate and during my time I had so many persons who couldn't participate and I became enemies to them because other persons told them the good messages and the good deeds and the...gbogbogbo [Everything] that they got from the whole thing and they were like oh, Tewo that is bad of you, so you didn't invite me for this particular survey and then another thing that I my question is because I wrote it out here, some of them will like after testing themselves...they know their status and it's really very difficult to accept it, they live in denial and by the time anyone calls them to ask did you test yourself, did you do test, some of them will say yes, some will say no, I have not done the test, ok I have done the test and what is your result, some of them will lie, ok I am not reactive, I am reactive or some of them will just keep quiet and it's a challenge because the major findings or what the implications of the findings is not even achieved, so that is my fear*

***KOL FGD_01***

*TEWO: …they said referrals to other facilities...I still think that messages could help out like during my own time, I had different persons who apparently worked with me as in participated and we worked things out, I apparently had to work with the numbers, call them even before the baseline...got to interact with them and also talk to them, get to know who they are, I never got to know all of them and I can't know all the community members, you can't know all like he said, it's an eye opener to the community because Jennifer said there are some people who you wouldn't even know and some you know, so I apparently had to dive deep into the community and then try to pass messages to them one on one and some that I can't reach, I send message to them and made referrals and references, I think that was all*

***KOL FGD_01***

*DOYIN: ...because you discover people don't want to meet up again due to what happened so it took extra effort from the KOLs here to say we need you and we promise you it'll just be two of us or three of us and then I remember when, when we started the endline it was meant to be the RAs direct to the participant, Ebun was to call and book and then she recorded that it wasn't as easy and the moment she brought in the KOLs to do the intermediary, everybody started coming in and that's because we had to establish that trust that ok, what you think would happen or what happened to the forty-two, don't worry, you are safe, you are calm. if I can be there, you can be there as well because I also would not put myself in a position of danger, so I think that's the effect of the KOLs…*

***KOL FGD_01***

*DOYIN: …so record has it that some months ago a group of people were arrested at a hotel in Ikorodu, Lagos, they discovered about seventy people were in the gathering of MSMs and forming different activities, but there also was the HIV test occurring at that time, but they got there and they arrested all of them even the hotel manager and this place has been known to host a lot of parties for a very long time, so they arrested them and then they said some of them would be granted bail if they pay like twelve thousand naira, so a lot of people paid the twelve and they escaped and then the remaining forty-two were the ones that had to be transferred to court in which they were sentenced to different bail conditions, rehab and all of that, so it was just recently that the last person was free or met the bail conditions through the help of NGOs and all but the thing is their pictures were posted online, their pictures were on social media, newspapers, TV and then another article came in and said most of them were HIV positive, so this is not just the stigma of coming out to your family and friends, also having to face that you are tagged positive, so I’m sure it affected a whole lot of families, a whole and people had to relocate from Lagos, people had to go. I know of three people that have moved to Enugu and then their life is not the same again, so most people have seen this thing and don't want to face conditions and that's why parties are now planned carefully, different locations, more strict measures are taken to make sure safety is ensured...*

***KOL FGD_01***

#### Distribution Issues (facilitators and barriers)

#### Facilitators

*Mr Gideon: Yeah! Social media was also useful to me because I was able to use, leverage on that part to talk to most people I have on my friend list*

***KOL FGD_02***

*Gideon: I was able to use my Facebook and my WhatsApp on people that have been friends with. But, I really do not talk too talk to but I know they really do not want to mingle when they go out for HIV testing. So, I was able to tell them about what the survey was all about, the self-test kit was all about and which many of them was interested in it. That was how I was able to mobilize them for the survey.*

***KOL FGD_02***

*I: What of using any material, were you able to use any material to reach out to them IEC materials?*

*Gideon: I have to start talking to them, leveraging on condom, that you will get free condom, lubricant and telling them more about HIV, and what it's all about. Most people are really interested doing it, most people do not know what it entails like you can get it effected through different means and getting the STI, so they just felt that HIV can be gotten through sex, they did not know the actual kind of sex, The feel ok when someone is physically well, they feel the person is ok, but I tried to explain things to them.*

***KOL FGD_02***

*Ikenna: talking about the stipend, I think it played a huge role in the study. Because, most of the time you get to find out that client will complain they don't have transport fare or one thing or the other. But, when we tell them about the stipend, not really like telling them that you are been paid to come and collect the kit, we just tell them it's your transportation, they see it as oh! since my transport is going to be covered it's good to go. It played a huge role, I don't think without the stipend we would have reached the mount of people we got to reach*

***KOL FGD_02***

*TEWO: ...apparently went a long way but the... I think they tried because if you could remember [Looking at Doyin's direction] you said something like for people to do the services you have to give them certain particular incentives and the incentive were kind of attractive, the pack itself, [Jennifer repeating after Tewo, showing sign of agreement] it looks fantastic and again because the MSM community are fashionable and the whole thing look so "sengemengish" [a slang for something fashionable] and it went well*

***KOL FGD_01***

#### Ease of recruitment

##### **Competence**

*DOUBLE D: ... I was able to convince some of them that I’m not trying to intrude in your privacy and whether you use the kit or not, it's not important, just accept the kit, I’m not asking you to tell me your status after using the kit, anything that...if it's your result is your personal issues, there are hotlines there that you can call and if you are not free coming to any TB, any of the MSM centre, you are free to go to a general population, a community health centre to access care, I even went as far as some of my clients that are living close to Igando, I had to go to Igando to drop that our short card talking about the CHC, to the heart to heart centre that in case you see anyone coming with this, just know that this is what is going on and they were able to...ok, no problem.*

***KOL FGD_01***

*JENNIFER: I will say the KOLs again because they are the face to the community, they are like middle person, eh, eh, they are the middle person between the community and eh... the organisation, the project either, so any one of it, so if they are not KOL because many they are our friends, we know these people very well and the information they pass to this people help a lot because if I should pass a wrong information, the person, I know this mouth dey fly, if I pass a wrong information, if I say another thing to another person and change all the whole thing and spoil all the whole thing because we pass the correct information to this people makes them to want to do, to want to participate in the study...*

***KOL FGD_01***

##### **Security**

*DOYIN: ...because you discover people don't want to...meet up again due to what happened so it took extra effort from the KOLs here to say we need you and we promise you it'll just be two of us or three of us and then I remember when, when we started the endline it was meant to be the RAs direct to the participant, Ebun was to call and book and then she recorded that it wasn't as easy and the moment she brought in the KOLs to do the intermediary, everybody started coming in and that's because the...we had to establish that trust that ok, what you think would happen or what happened to the forty-two, don't worry, you are safe, you are calm. I’m your...if I can be there, you can be there as well because I also would not put myself in a position of danger, so I think the... that's the effect of the KOLs and then, I don't know, trust...*

***KOL FGD_01***

### Success stories

*A-Z: The two success stories is that I think that I have which am very happy is that the kit is here and some ... the five people I said were reactive they have not accessed there HTS before, which I invited them and they know there status and they are reactive and there success stories is that they accepted there result and I linked then to care, which they are now doing fine*

***KOL FGD_02***

*Is there anyone who has a success story to share as regards the distribution, what enhanced your story?*

*Ikenna: I will take what... like what helped me in succeeding... social media was a huge, it was like a ... I don't know how to put it... social media helped me and my network. By the time I get to tell a friend and then my friend tells his friend...the main thing is while telling friend or anybody. Just give them the eligibility criteria, it made it easier for me. So, at the end of the day, when I get to invite one friend for the study, he comes with four other people knowing the fact that he has let them known about the eligibility criteria. It was left for me to do my little screening and then they are all there for me.*

***KOL FGD_02***

*Raphael: What enhanced our success story was the stipend. Where by you want to convince somebody to come for something, you know (explained), what you will here is that "me I don't have time oh! except they are going to give us money, or if they are going to tip us up or if they are going to give us anything. So, my own success story was mainly the stipend, because it motivated most of my client to come.*

***KOL FGD_02***

*…I was about to talk about the video you know, if am to send the video to my social media, Facebook and WhatsApp group. I created a group, I have some few MSM, most of them watched the video and they were like self-test! (he expressed surprise), that they will like to you know... the video, it was a success.*

***KOL FGD_02***

### Challenges

*DOUBLE D: some of them they don't like identifying with any TB [Slang for MSMs] gatherings, most of the MSMs that I have they don't like identifying with any TB gatherings*

***KOL FGD_01***

*JENNIFER: talk about the funders that is giving out, that is giving us this medication for the people living with HIV, there will be a time that they will take it but trust me there will be a time that everybody will die because nobody will want to buy drug trust me. See I have been working, I have been working as a peer educator for good eight years, so I understand the community very well, see nobody will tell you that they want to buy HIV test kit with money, is a lie and why is like that, is because majority of organizations have spoil it all by sharing money to this people, they've been giving them money, just like you have been giving me money to do test before, then you are now telling me that this same HIV test that you are giving me money for, now there's a test kit for me to buy...I will not buy it because we still have some people that they will tell you that even though after the HIV test that they will deny that they don't want...they are not HIV positive, they don't want to receive any medication, they don't believe in your test, they don't believe in anything, they want to remain like that, they want to be fine like this, they are okay, they are fine, so if they know that they will be buying this test kit, they will just tell you that, please sorry, hold your test kit, I’m not doing because I’m okay*

***KOL FGD_01***

*Ikenna: The common question I heard was if they are going to disclose their status to us and which I told them NO! That it's their decision, that if they want it or if they don't, and the complaint I got on a regular basis was the timing. There was so much time been spent with the RA's.*

***KOL FGD_02***

*A-Z: I think I have few people that when I introduce them to the oral self-test, they now said but, HIV can be found inside blood but why the gum. That is to say, I can get it from kissing, I now explain not that, but it is your anti-body that we want to check, so that your anti-body will now lead us to where we are going, so they now said ok.*

***KOL FGD_02***

*Gideon: ... I want to talk about the challenge we had, that's because we were given some certain target to meet up to some range of age, and later we had to cut off that age because they felt we are recruiting them too much and we actually have to recruit people older, from 40, 50 years and it was really hard for us to get, why it was hard for us to get at that point in time because they didn't tell us at the beginning stage it was going to happen like that because we were all leveraging on that particular age, which was given to us at that particular time. So, assuming we were told ahead, we would have leveraged on the network we had and try to link out to older community members to reach to them too for the self-test.*

***KOL FGD_02***

*TEWO: ...what about the security of the KOLs on field or the interviewers on field, what if something happens to them or there's a particular crises or chaos on field, what security measures would be given to them that's just my own.*

***KOL FGD_02***

*Personally, the only challenge I had was the fact that we have different age group to recruit; we had the teenagers, we had the early young man and we had the old men. Now when we look at our cycle or network we are more likely to recruit our age group very well, it got to a point we were been asked not to recruit our age group any more that we need to go for older people and the under age. That was a big challenge bringing elderly men down to the clinic or going to meet them at their own convenient time, would they really accept it, would they really accept what we want to talk about. So, it was a challenge actually to us or should I say to me personally.*

***KOL FGD_02***

*The challenges we had was that like he said, most of our client which we reached, or people of the age which was given to us for us to reach, which we did, which they said we've really exhausted, that we should go further looking for people of high class and working-class persons. But, we are able to reach them and again we were not able to reach some of them because most of them are always at work and they could not allow external persons to come to the office, they don't want other people to know what actually they are coming for to do. So, at a period of time, I think most of them there schedule was really tight to come back. Their schedule was really tight for us to reach out to them, that was just my challenge.*

***KOL FGD_02***

*A-Z: All my personal challenges are the age, like I said earlier on, one of the major problems for me is that of recruiting the elderly, the high class. Because, I notice that I don't mingle with high class people, I mingle with my normal range of my age, the adolescence, it comes to a time that I have to pause because I cannot reach those high class. Because in this life, power ends where you can reach, that's my own challenge.*

***KOL FGD_02***

## HIVST & MSMs

*I: how important is it for MSM in Lagos to have access to HIVST?*

*Ikenna: it's very important because I should say that research has it that HIV and MSM are like (he clapped his hand, demonstrating bond) brothers and sisters been the fact that... because they feel it's male, same sex, and then there is nothing like one getting pregnant and all, they are likely to have unprotected sex, for getting the fact that you can contact HIV and STI's and then you get to find out that because of certain friends are this close and that they know themselves as MSM they get to have sex with each other, most times unprotected because they trust each other which is really wrong or because one is in a relationship and they are discrete. So now providing HIV self-test kit for them is very important being the fact that... there is something called anal warts; it’s a virus but I will classify it as a sexual transmitted infection…*

***KOL FGD_02***

*A-Z: It is very important! I will want this test kit to come to Lagos so that, the test kit can bring the goal of the 90: 90: 90 to Lagos state. Because if the test kit is available people that are not willing to do there test by using the normal way can go into the pharmacy and get this test kit and do there test and know their result. I fit is positive they will have to look for a place of where they will be using, And from there the goal of 90: 90: 90 will be achieved because 90% if we have this test kit in excess, surely 90% will be tested, that 90% the second 90, 90% those people that are tested will be led to care, then the third 90 will surely have the viral suppression then we will achieve it.*

***KOL FGD_02***

## Preferred distribution channels

### Health facilities/anywhere

*I: And of course from CHC here you don't mind getting it like you said*

*R1: Yes!*

***MSM_IDI_01***

*R4: Second option I can come here (CHC) to get it*

***MSM_IDI_04***

*R4: Like Primary Health...just may be somewhere just close to me like, somewhere I'm comfortable with*

***MSM_IDI_04***

*R9: And through perhaps the hospital*

***MSM_IDI_09***

*R 12: I think the best place will be from health centres*

***MSM_IDI_11***

*I: like where would you want to go get it, where would you want it to preferably be for you to go get it?*

*R 18: Clinic, health centre*

***MSM_IDI_16***

*I: If it will be sold, where would you think will be the preferable place to make it available?*

*R 10: Maybe any of the centres!!! Your clinic*

***MSM_IDI_20***

### KOL

*Gideon: the KOL were able to reach different kinds because we've been able to leverage our personality with the community. People see us as stakeholders, the community see us as stakeholders and we've been able to create that connection with our peers and our friends which we are leveraging on for whatever research we may want to do with them, because we feel okay each one person is an advantage to whatever research we actually want to take in place, whether they are your enemies, whether they are your friends as far as they are community members, Because each information you want to pass out really will help the community. And as a KOL, we’ve been able to accommodate all kinds of people, that’s why we are been there, that's why we are stakeholders. We've been able to talk to them, we've been able to put our priority to make them understand that we are there for you people and we are the same as you and we standing in the same community. So, we are able to leverage on the part that they know us and we are able to reach out to them and give them the right information of about what it is all about and give them the right protection and what protection is all about…*

***KOL FGD_02***

*R1: From a KOL, fine*

***MSM_IDI_01***

*R2: The KOL, no problem*

***MSM_IDI_02***

*R4: the best way is through Doyin (KOL)*

***MSM_IDI_04***

*R7: I would want to get it from the key opinion person…*

***MSM_IDI_07***

### Others

*Raphael: Beyond using the KOL, I think it should still be the social media, because I believe it is not only the KOL are used to phones and social media, so social media with the video...*

***KOL FGD_02***

*Gideon: I fell not just leveraging on the KOL, I feel we have influential people in the community that have so much network, that have expansion of network which we can leverage on them also*

***KOL FGD_02***

*FGD 2****:*** *You mean not basically community members*

*Gideon: ...community members which have network with community member’s, if we are to reach just community members which we know that there are some people who are not community members, they have connection with the community.*

***KOL FGD_02***

*R2: I even prefer the way I even got it, it was ok by me than going around, I even like it, you can give me again even though more and more*

***MSM_IDI_02***

*...coming here is not far because school is close by ...getting it from the leader as well is still good because in the sense that if...you and your leader can meet, like for example your leader stays somewhere like Ikorodu and you stay at Ketu, you guys can decide ok let's meet at mile 12 or something, just pass it on than you coming all the way down to Yaba or something, so I believe that giving it to team leaders or anyone is very very good as well, it helps and it reduces the stress in getting it as well for someone that is far but for someone that is close by, you can still come down here to pick it.*

***MSM_IDI_03***

*...some NGOs also...*

***MSM_IDI_05***

*like community centre here*

***MSM_IDI_10***

### Pharmacy

*A-Z: if there is a good pharmacy that you can go in and pick it up. Either you buy it or in another means…*

***KOL FGD_02***

*If they are available in pharmacies and everything, it's even good because the more closer to the people, the better the people get...*

***MSM_IDI_03***

*May be like a reputable pharmacy that may be you know they have these stuffs and they, whatever means, whatever form they are giving it out, either free or what...you can get it from them and yea, it would be, I don't want to say confidential but it would be like really easy to get it so may be good pharmacies, from this place, the opinion people and you also asked if I would like to buy it*

***MSM_IDI_07***

*R8: By now it should be in pharmacies and chemists, so that you can just get it*

***MSM_IDI_08***

*R 12: I think the best place will be from pharmacies …*

***MSM_IDI_11***

### Supermarkets

*From a supermarket*

***MSM_IDI_01***

# LINKAGE TO CARE

This describes pathways for enrolment into care by participants who used HIVST kit.

## Barriers to linkage

*So after testing you were just fine, you didn't think you needed any other thing*

*R7: I think probably may be because of the distance of where I was to this place*

***MSM_IDI_07***

*R7: I would probably want to come in but then, I would, it would be very, I would be very particular, not particular but I would be very sensitive about whoever I’m talking with, whatever the result might have been, I will be very much sensitive… I mean I've not talked to a whole of lot people but I think if I knew that this person is a very open person like...just take whatever you say, I would come in but then there is so many people with different personalities and I don't really know so much people here, so there's a little bit of trust, mistrust issues…*

***MSM_IDI_07***

## Confirmatory test

*I: But did you bother to go for any confirmation test after that?*

*R1: No*

***MSM_IDI_01***

*R 2: I didn't go for confirmation test... it didn't cross my mind, what is in my mind is checking my result, for this it's just new to me now, for this confirmation test… what could actually make for me to go is when I checked my result and my result is not what my mind is saying, I would like to go, because what you are not expecting because it is coming up, you have to cross check whether it's true or not, That's what can make me to go for it*

***MSM_IDI_02***

*I: After testing did you bother to go for a confirmation test?*

*R3: No, I didn't… because I believe the result is accurate and there is no need of me going anymore*

***MSM_IDI_03***

*I: did you bother to go for confirmation test?*

*R4: I just did*

***MSM_IDI_04***

*R6: Yes I did a confirmatory test*

***MSM_IDI_06***

*R7: ...the result was accurate because before then I had come in to the centre for a test and so the result was pretty much like confirmatory and it was, it was good*

***MSM_IDI_07***

*I: so that's the only thing, did you bother to go for a confirmation test?*

*R8: No, I didn't, May be time or something, I don't know why, I didn't bother because it's negative*

*I: so is there anything that would have persuaded you to go and do a confirmation test?*

*R8: Ok, maybe I heard a news or I heard that the kit doesn't work well or is not giving hundred percent accurate...I would have said ok, I won't trust the result and I will go for test*

***MSM_IDI_08***

*I: Did you do a confirmation test?*

*R9: Yea, I did*

***MSM_IDI_09***

*R 12: I wanted to be sure it was really accurate and not be like error before, I have heard such results, but I was told that it was pretty accurate and I came and I did back up test and it was also accurate.*

***MSM_IDI_11***

*R 14: I wanted to know, I was anxious to know since it was not giving me straight result, I have to quickly come for confirmatory to know my result, it wasn't giving me what I wanted, I came the following day, I think I called Ikenna, Ikenna told me to still come for confirmatory test.*

*I: You feel the KOL was very useful for you to come for confirmatory test?*

*R 14: Yes*

***MSM_IDI_13***

*If I were positive, like I have actually said earlier, I would have been here say ok let me have a confirmatory test or something like that.*

***MSM_IDI_16***

*R17: O yes! I worked into TIERs and I did another… After using the self-test kit*

***MSM_IDI_17***

*R19: …I had to go and do a confirmatory test...* *It was negative... but like I said I wasn't or didn't actually trust it like you know cos I've done a whole lot of risky things, you know, this year, so I was like can I trust this kit, so like I said I had to go and do a confirmatory test before I had a complete rest of mind you know*

***MSM_IDI_19***

*P.T 1: Yes, I did go for the confirmatory test at community health centre*

***MSM_IDI_21***

## Linkage channels

### Helpline counselor

*I: Did you get every information you needed from the hotline?*

*R 12: Yes I did, She told me I will come for a confirmatory test and I will be given free drugs, I will be well taking care of basically so!, it was good to know that people had my back, even people I didn't know, they had my back and it was quite good, I was quite appreciative*

***MSM_IDI_11***

*I: Yes you didn't call the hot line, but do you think that this hotline is necessary?*

*R 16: Yes, I really think it's necessary*

***MSM_IDI_15***

*I: but do you think the hotline is something you would recommend for future self-testing programming?*

*M2: Yes! very much, because at some point if you get stocked, you need to call someone and because this is a very delicate topic, HIV...you won't want to call someone you know because you wouldn't want the person to start digging in and probing to know your result or whatever, so I just think the hotline is very ok, so when you call the hotline the other person at the receiving end being the nurse or whoever will attend to whatever pressing questions or need you have.*

***MSM_IDI_22***

#### Called on their own

*I: Did you call hot line?*

*R 12: Yeah I call, the hot line immediately after the test, and it was during the weekend and I was told to call back on Monday*

***MSM_IDI_11***

*R17: I made a call to the centre after the test but necessarily to numbers that were on the instructional sheet.*

***MSM_IDI_17***

#### Were called by help line counselor

*M2: I don't think the instruction say that I should call back, so I wouldn't have... but if the instruction had read that I should call back, I would call...*

***MSM_IDI_22***

*R6: ...no, I did not but the hotline called me...so, I didn't feel the need to call*

***MSM_IDI_06***

*R9: But they called me... It was surprising because they kept on calling me to come and do the confirmatory test, so it was really nice*

***MSM_IDI_09***

*11: No! They called me and I told them about my result…*

***MSM_IDI_10***

*R 13: No! I didn't think there was any need because I was hoping that during the endline survey, maybe be asked and I will have an opportunity to do that. Yes, they called me once and asked me how did I feel using the kit, and I gave a good report, although that day I was in a hurry, I was not in the mood of talking that day for a long period. She asked would you be comfortable sharing your result with me, although it was negative, I said no, and the reason was that, I was with the phone in a public place, I didn't want to be saying that I was HIV negative, or positive in a public place. It wasn't about the hotline. It was about where I was. If I were in my house alone and she called, why not?*

***MSM_IDI_12***

*I got a call from Ebun, I think some days later if I used the test kit and I told her yeah, she asked if I care to share my status, I told her yes, obviously if it were positive I would have just kept her quiet.*

***MSM_IDI_18***

*R19: No, I was called, so I had to tell them there was no need for me to do the test because I had done it like a week before, I had my result with me, I just showed*

***MSM_IDI_19***

*M2: ... I was called, yea by the nurse counselor, I was asked if I would want to share my result which I did...*

***MSM_IDI_22***

### Key Opinion Leaders (KOL)

*generally, even the hot line, even my client was like ah!... the time I called my client are you not going to come for the, he was like you are still calling me to come, he was impressed. The follow up was ideal.*

***KOL FGD_02***

#### Called on their own

*And after they conduct it by themselves they called me back that this is my result, the one that was reactive to it called me and what is the next step and I now said ok, go for your confirmatory test and confirm*

***KOL FGD_02***

## Post-test counseling

### Sought for post-test counseling

*I: Now you think that counseling is necessary?*

*R18: I think it's necessary, she did her job, she did a good job, she counseled me well*

***MSM_IDI_16***

*I: did you seek any form of counseling after testing?*

*R4: Yes*

*I: From where?*

*R4: Who else, Doyin*

***MSM_IDI_04***

*R 15: Like them talking to me... I came back here to pop council. I called her immediately, so she now told me, the steps, how am going to do it, that I should come this day, this day, that she is going to attend to me... at first she asked me questions, how it happened, how I see it. But, first of all when I called her she asked me if I could, like tell her just brief her how it went (how you ran the test), I told her, she said it's ok no problem that all is well that I should just come... she will give me the drugs, I said ok and I came, and since that day, I have been taking my drug****...***

***MSM_IDI_14***

#### Reason for seeking post-test counseling

*R11: …I come here (Pop council) because this place is confidentially secured, that is why, without that I wouldn't go to any other community facility.*

***MSM_IDI_10***

*I: Now, after using this test kit, you said you came for the post-test counseling, they told you series of things, now, you have any challenge of coming to this post-test counseling from your heart?*

*R 14: No!, I wanted to know, I was anxious to know since it was not giving me straight result, I have to quickly come for confirmatory to know my result, it wasn't giving me what I wanted.*

***MSM_IDI_13***

### Did not seek post-test counseling

*I: did you seek a post-test counseling?*

*R 2: No!*

***MSM_IDI_02***

*I: So after testing, where and where did you seek support or post-test counseling?*

*R7: I didn't*

***MSM_IDI_07***

*I: But apart from the fact that you were called and you came for a confirmatory test, did you on your own seek any post-test counseling?*

*R9: After the test, no*

***MSM_IDI_09***

*R 13: I didn't go*

***MSM_IDI_12***

*I: Now you didn't go for any of these things, what could have persuaded you to actually go for it?*

*R 16: (Deep sigh of relief) go for counseling after testing yourself. My own kind of person oh! Nothing oh am sorry!!! I rather go online and read it.*

***MSM_IDI_15***

#### Reason for not seeking post-test counseling

*R2: ...am not sure, if the person likes it, maybe it's for the person, but for me it's not necessary. Because I tested negative*

***MSM_IDI_02***

*R6: ...no, like I said the internet has always been my friend, it has always been an ally for me, so I didn't...I just brushed up on my knowledge on what to do if but I didn't really think there was a point. Regardless of the result I don't think so, if I were positive, yes, definitely but if I was negative, I would rather not take up the space that could be given to someone who actually needed it*

***MSM_IDI_06***

*R7: The last time I had like a counseling thing, even if it was not like I was heartbroken or something but the response, I mean you know if you are negative, it's just like a second chance, you have to be more careful and then if you are positive, you know you also be more careful and but then the reaction was just... I didn't like it, so I can't even think of coming, because they already told me...*

***MSM_IDI_07***

*I: After testing, what would have made you go to seek counseling after testing?*

*R9: That's if I was positive*

***MSM_IDI_09***

*The basis for going for counseling is dependent upon who the person is if your privacy will be highly respected, if there will be no input of fear in me, that's it. Help me, don't destroy me the more, that’s it…*

***MSM_IDI_15***

*R17: There was really no need for a counseling, I feel you get counseled when the result is not necessarily favorable, so you get counseled for okay what's the next step, this is what I've seen, how do we move forward from this and you know.*

***MSM_IDI_17***

*I: So why didn't you seek for a post-test counseling?*

*R 20: Ah!!! Because I just felt confident about the result and then I had the fear but then again I was like let me just seat and wait for the three months window period*

***MSM_IDI_18***

### Follow-up calls

#### During study

*JENNIFER: Follow up the client, be at that point you gave the client the kit… I can remember we met a client that says that he has not used his kit that he still hang it inside the room, I was like my dear use this kit, he said, I don forget how dem say make I use am, upon say the thing them put am on top paper inside the this thing, you understand so most of them, I can remember that I helped about five people, I just went to their house like how far, have you used the kit, so how was it, do you like it…*

***FGD_01***

*JENNIFER: They are our friends, even if they are not your friend, make sure you know someone that is their friend to take you there, but you are a KOL, you are just like the face of the community, so try and go to their house, know how far, know how they take use am, some of them fit leave am for house, them fit dey fear, so if you cannot go, you no fit know wetin you wan tell the person to calm am down say if you use am nothing go happen, I use the kit for about three people for myself, I was like oya bring it out let's do it, oya do it like this, do it like this and all of that, you understand, others are still scared because that's their first time and they have been engaged in different sex and all of that, so they are still scared of them using it now, they will now know that they have HIV, they will just die, so it's still need this follow up of a thing.*

***KOL FGD_01***

*R: Like being informed of ok even though you know your result, the steps, the prevention, what you can do, the dos, the don'ts, just counsel…*

***MSM_IDI_05***

### Friendly services

*JENNIFER: Another help I think is that if there is any client that find out that he or she is reactive and he don't have the time to come to the facility for the drugs or for confirmation test, even if we can just take it out to them and do all these for them on field and still help by giving them their medication in their own private house. Because I have a lot of people, I can remember in the past, I used to have this particular friend, a particular group of people that we do go to their place to deliver their drug for them and trust me they are really doing well with it…*

***KOL FGD_01***

*I: And you liked...your discussion with the counselor...was it fine for you or what do you feel...?*

*R9: She was nice enough, before they gave me the HIV test kit, they've said it already that HIV is not a death sentence, so just in case you are actually positive, you come to the hospital for counseling and treatment*

***MSM_IDI_09***

*What persuaded me to come is because I have plans for my life and I don't want to put it to waste, and an aspect of me thinking of that, I have to take care of myself very well. I was intrigued by what Miss Ebun said that she know someone who has lived for how many years now and he is still kicking, working very well and he is healthy. That is a good support, that's a good word of advice people will love to hear****.***

***MSM_IDI_14***

### Quality of care

*TEWO: Counseling, they will need counseling, proper counseling and psychosocial support before and after using it, they can have that support in case for those who are reactive they can have linkages or referrals to other facilities and again because of the mental health issue associated with the epidemic.*

***KOL FGD_01***

*Everything I needed was available, it was when I came back here they even did the test for me again, they took my blood. So, I was really comfortable giving it to them.*

***MSM_IDI_14***

## Visited a facility

### CHC (Pop Council)

*R6: Population Council...to do a confirmatory test... I was given two self-kits and I used the second one...just to make sure*

***MSM_IDI_06***

*I: ...you said you came to the facility at some point?*

*R6: Yes, after three months*

***MSM_IDI_06***

# RECOMMENDATIONS

This section describes participants’ suggestions on how to improve future HIVST use and distribution.

## MSM Tag

*DOYIN: One other recommendation I would is to remove the tag MSM...*

**KOL FGD_01**

## Capacity building, Client recruitment & Distribution

*A-Z: …by calling them, visiting them because they are nearby to me, I visit them and encourage them not to lose hope and to talk more about the test kit and even HIV on its own... I noticed that him on his own, positive client, you need to get close to the person…*

***KOL FGD_02***

*It depends how we the KOL we recruit. Like me, the first day I started writing something on my Facebook, about HIV, prevention, which people said ah! ah! why are you writing, sending like bulk SMS to people about HIV, I want to get something from them. So, they now started chatting me up privately, that you are writing so so so!!! I will like what you are writing, how can I access this thing? From there someone that is willing to access, I will now first of all tell the person that there is transport for him… that is your transport, thanks for coming.*

***KOL FGD_02***

*Ikenna: The only support I would have needed is my fellow KOL probably helping me when am trying to meet with some particular set of people, probably for example; low class people. The ones who speak Yoruba or pidgin, am not really fluent when it comes to that part. So, it got to some part when I told them about the eligibility criteria, even when they don't understand it, I make them to come here, I get to direct them to a friend, a KOL, who is also recruiting, since I can't handle this person, can just take over, and if he takes over and sees that he is actually eligible, he gets enrolled. So, I thought if we were being given a target, not personally, but a target to all the KOL and make it look like you all should work together to achieve this goal but then you will still earn the same thing, it would have been easier and we would have been able to meet all sort of group; the high class, the low class.*

***KOL FGD_02***

*…we had people from the low class that had language barrier which let's say they were eligible at some point in time from outside but it got to a point which they got to the interviewer and they were like language barrier, that most of them could not understand the questions they actually are asking, that was the thing... for us to reach our target there are so many people that do not understand so much English. So, we really need to put some people inside it that will be able to speak out to them that really understand that both of them can really understand each other for those of them that are not actually fluent very well with English.*

***KOL FGD_02***

*Gideon: To improve on our capacity, I felt more incentives should be given to us, the KOL to improve our capacity and to ginger us more on how we mobilize our people, and more training and awareness, that's what we need.*

***KOL FGD_02***

*A-Z: When a survey wants to occur, I think people that are involved are supposed to think of the people they will recruit...these are the KOL that are supposed to recruit adolescence, these are the KOL's that will recruit the older ones. Like I said earlier on, I don't have power to recruit those elderly ones because I mingle with my class, you cannot see me playing with elderly person, I mingle with my age. Maybe we have 20 KOLs, 10 KOLs should recruit so so so age while the other set should recruit so so so age, so it will be balanced. And another one is training, more training on this oral self-test, so that people will know more about it, if this thing can go viral, I think people will quickly accept it than the normal test kit.*

***KOL FGD_02***

*Raphael: I will also like to talk more about the training if there is any to support the KOL you know, so that if they will direct the training for the self-test that will improve our knowledge that will also help us to know the level we will recruit our clients.*

***KOL FGD_02***

*If this study is to continue, we the KOL are supposed to improve in knowledge about the test kit, whereby we want to talk about it, we talk with confidence, bold, I believe we will get more...*

***KOL FGD_02***

*We didn't emphasis on older people, how you can work up to elderly people and tell them about the self-test kit. We need more training when it has to do with approaching elderly people. There is difference between high class and elderly people, people in there 40, 50, 60's the way I approach them wouldn't be the same thing the way I approach my friends in there 20's.*

***KOL FGD_02***

*Gideon: …so many people during this study were not able to be reached for the self-test kit, who really wanted to participate but were no so much time for them to participate because of the short period of time and I feel since the test kit is going to come back, we should not just target the community...but if we are to look at the community alone, we should leverage on the part of reaching out to more people. It's just like you connecting to someone and another person connecting to another person, we should always leverage on the fact that we should be able to get so much people and give out so much information about it, so that the test kit will be able to circle it and it's able to reach out to people who does not have means to reach out to.*

***KOL FGD_02***

*I: In the future do you think that this text kit should be distributed?*

*R 12: Yes I think it should be and I also think as you are distributing it, it should be like a clinic or a health centre where they should be referred to, and they should be referred to someone not just giving them test kit and not just knowing what to do after they have gotten their test…*

***MSM_IDI_11***

*I: Now in the future, do you think this self-test kit should be distributed?*

*R 14: Yes, it should… Through hospitals, local government, communities, schools*

***MSM_IDI_13***

*I: Would you prefer the KOL giving it to you or you walk in somewhere and access it, how would you want to obtain this self-test kit?*

*R 14: I think the KOL should be the one to…*

***MSM_IDI_13***

*Well, if you guys could just go to rural areas and distribute this from people to know more about it, not just us coming in here, I think that's better*

***MSM_IDI_16***

*I: Are there other medium we could use to distribute this?*

*R18: Social media, yeah! I actually have a platform where you can actually distribute it and all*

***MSM_IDI_16***

*R 20: If there is a spot, maybe a hot spot or somewhere that they know they distribute, this is the where they distribute this stuff…*

***MSM_IDI_18***

## Future uptake

*He was like he liked the study, he wished that the study will continue, so that we will reach some people out there so that they can have the opportunity to come into the study the way he had the opportunity*

***KOL FGD_02***

*I: Why do you want them to use it?*

*R 2: …they have to make use of it because some of them do not know what they are doing... some of my friends because of this HIV they killed themselves, because of this HIV they are indoors for so many years, so I want them to use it.*

***MSM_IDI_02***

*I: You already said you gave one of the kits to your friend so it's something you would love to recommend to other people*

*R3: Yes!*

***MSM_IDI_03***

*R6: Yes! It’s actually something that I actually recommended to all my friends who are of the LGBT community.*

***MSM_IDI_06***

*R7: …it'll be nice for them to try cos these same men would have it, secretly consult people who don't know what they are doing or who don't have an idea of what it is or who would rather lie to them and get money off it by telling them they are ok and then tell them to go and do some crazy things like the one they do like sleep with virgins, harm people, kill this, kill an albino, just because they are not coming, they don't have the right material to even test themselves. I would recommend it to those kind of men, recommend it to housewives that are scared, I will recommend it to anybody of any sexual orientation...*

***MSM_IDI_07***

*R 13: Yes! I think I will want to recommend it to others because I feel something like this if people are aware of it they will appreciate it.*

***MSM_IDI_12***

*Well, my reason to recommend it to them is that one: I want to help them out to know there status and as I told you, we are always there for each other, it's best for you to know your status early…*

***MSM_IDI_14***

*…this should be accessible as well for people that like privacy to have themselves tested and to seek the right care…*

***MSM_IDI_15***

*everyone should use this, I really want you guys to go to all these rural areas and others and distribute this stuff so they could get it because if you ask them to come for HIV test and all that they wouldn't, they will be like no, I don't want to be coded and all that, I think this is better, I feel good about it*

***MSM_IDI_16***

*P.T 1: Yes I would love to use it some other time.*

***MSM_IDI_21***

*I: …is it something you can recommend to other people?*

*﻿M2: ﻿Yea! Sure! Because it's fancy*

***MSM_IDI_22***

### Number of kits

*R7: for me I think one is fine and if, if may be the person wants another one, the person should also be given and may be there may be a procedure to get it but I don't think it should be so stressful getting another one, otherwise they should just give two but if, at least maybe they should say the maximum you can get it two but we'll give you now to go and use it and if you feel you want, and if you feel you want another one, we would give it to you but that's like the limit and if you want more that means the process can now be may be because you don't want anybody who is getting it to go and make money off it, yea but initially I think one, one is fine.*

***MSM_IDI_07***

*R6: I think it would be better to give people two and if it's going to be distributed. If it is going to be on sale, let them know this is where they can get it and at what price, let everyone know, so it can reach everyone but if it's not going to be on sale, then it's best to give people two, give them one and then tell them about the three months wait, so they use the other one after three months but if it's going to be on sale, then just give them one, give everyone, as much people as possible and then tell them where they can get another one.*

***MSM_IDI_06***

*R4: ﻿...I would say you can give as many as possible*

***MSM_IDI_04***

*R1: ﻿If I can have the one that will be enough for me for a year, I'll keep it in my house*

*﻿* ***MSM_IDI_01***

*﻿R8: ﻿I don't know, looking at the size, it looking a bit big... may be let me say the person can have just three*

*﻿****MSM_IDI_08***

*R 12: I think you should give two, because sometimes if there is error you can also confirm and then someone like me that is positive now, I just needed to be sure that ok I was really positive at that point in time I was really anxious, so two is needed, it's like a pre-confirmatory test, so two will be the best.*

***MSM_IDI_11***

*R13: I will suggest giving the level of education in Nigeria that people should have one every three months, they should not have more than one at a time*

***MSM_IDI_12***

*R18: Two will be ok, like what happened to me, maybe I didn't actually wait for the duration, so the thing didn't come out at all, so I have to like try the other one, I think two is ok*

***MSM_IDI_16***

## Helpline support

*I: Do you think the hotline is something you would recommend for future testing?*

*R9: Why not?*

***MSM_IDI_09***

*...I don't know but it's not really a big one because if the hotline is functioning and there are people on it 24/7, because I don't mind at night, I called her I wasn't getting through to the person…*

***MSM_IDI_10***

*Yeah I think you guys have your hot lines, exactly, you can actually reach out to, I think that's the best thing. Whoever tested positive should just call the hot line and the next thing will be done.*

***MSM_IDI_16***

*M2: ﻿...but if there's some kind of a toll free line that will be written on the kit, so even though the person is in Abuja, if the person travels to Abuja or whatever, would call, then the person at the other receiver end would tell the person what to do if the result is positive and how to take it up from there.*

*﻿* ***MSM_IDI_22***

*R 11: …just getting the drugs and constantly reminding me of the...*

*I: You just wanted a direction on how to get the drugs and a follow-up?*

*R 11: Yeah!!!*

***MSM_IDI_11***

## Incentives

*DOUBLE D: If there's going to be distribution of the kit at least to reduce the expenses, at least if I’m meeting five people, I can buy them drinks, at least secular friend does it, buy them drinks, buy them snacks and from there what is supposed to be going on is already going on… I am saying that the Funders of the oral self-kit can easily ok, we are giving you so so amount to support when distributing this kit…*

***KOL FGD_01***

## Instructions

*R19: A challenge...if someone probably is not well educated like I said if he can't read properly and adhere to instructions, the person may probably not...*

*I: How can we overcome that?*

*R19: May be add more languages like I said, Pidgin, Yoruba...*

***MSM_IDI_19***

*I: So we are maintaining that there should be a bit of counseling sentences on the pack...*

*[All echoes 'Yes!' 'Yes!']*

*I: ...so whoever reads knows, you know the first line of action*

*[All echoes 'Yes! yes!' and nods in agreement]*

***KOL FGD_01***

*DOYIN: ﻿…it comes with the videos because from what he said, it was very easy for me to assume the instructional sheet was very good and most of my people understood and there was really no challenge except people that were...failed to check and then I had to be on the phone and oya, are you ready, let's do this and then the whole twenty minutes of waiting we have to keep talking and when the results came out and they were fine and some people I had to meet, oya are you ready now let's do this, so I'm proposing helpful information, correct information because that's one thing I really liked about the training we had before the baseline, we were trained to answer different kind of questions in the exact ways and so that helped greatly. Now that you are negative, these are the things to do to continue staying negative, this, if you are reactive, this and this is what to do…*

***KOL FGD_01***

*﻿R4: ...to better it, I think the manual should have been written in more comprehensive way that anyone could actually just pick and understand it like first glance or something.*

*﻿* ***MSM_IDI_04***

*﻿I believe it should just be strictly kept for the adult, only for the adult use, so a child will not end up drinking the liquid. I don't know if it will be harmful or not, for children and all that, underage they can keep using that one (conventional test), or if their parents are there with them they can use the oral self-test kit*

*﻿* ***MSM_IDI_08***

*R 12: Maybe it should be put on Youtube or sent to the person’s phone, the person can use it as a preview if the person doesn't really get to understand the language on how to use it from the instruction guide…*

***MSM_IDI_08***

*R 12: I don't really get C and T, if they can use a sign like positive or negative, or an error if the thing didn't get it at that point, so it will be easier for people to understand, because of the two stripes and one stripe...*

***MSM_IDI_08***

*R 14: it should be clear, if you are positive or negative, instead of the invalid. It should just be clear or stated. It should just be stated, if you have it; you have it, if you don't have it; you don't have it.*

***MSM_IDI_13***

*I think there should be somebody in place to give them orientation for people that are not learned to use*

***MSM_IDI_13***

*If you can translate it to our local dialect, like maybe Yoruba, am Yoruba for example, if you can translate it to Yoruba, translate it to Igbo, Hausa, those three main languages, dialects in Nigeria…*

***MSM_IDI_15***

*I read that you are not supposed to like have food particles or crumbs in your mouth before you conduct the test, that means you must have eaten like forty minutes before conducting the test or you would have taken water before conducting the test, I think I read something like that somewhere, that if you don’t do so, it's going to alter the result eventually, so, I don't know if there's a way that it could be done to accommodate because some persons, trust me, may not actually read those instructions on the manual…*

***MSM_IDI_22***

## Linkage to care

*A-Z: Talking about the linkage that is the work of the KOL to link them. Because what I normally tell my fellow KOL... now that you are the one that is preaching something, you are preaching now, safer sex and at the party you are the one that is having unprotected sex. They will be looking at you that you are not responsible, you understand. If I say don't do this and this and this, I too that said that you should not do it, I follow what I’m saying, they will ah!! (take caution) this person said that we should not do this, ok, don't let us do it and he is not doing it. They will like to mingle with you and explain everything. And I talk to them and link them to the proper clinic, I have five that are positive, there is one that said that he cannot use this place, he has a family Doctor, he need to tell his family so, he will use his family clinic, I said ok go. I still follow up on him, he is on drugs now. So, it is the work of the KOL, if the KOL's are responsible, they will link the person.*

***KOL FGD_02***

*Gideon: I feel the KOL should do proper follow up to the client they are able to reach out to during that period of time, like making follow up, giving the correct information of what the self -test kit is all about and giving them proper information about the HIV test and HIV in general, and if we have to find out that one of them is positive and they do not want to use this facility, we should make out time to follow them and they do the right thing…*

***KOL FGD_02***

*Gideon: they should be trusted, they should create confidentiality between them and their client to link them to confirmatory test and post-test*

***KOL FGD_02***

*R8: a call should be made after, like if I'm giving you the kit and I have used it, I should be given a call may be in two weeks’ time to know how far if I've used it some people it's easier for them to voice it out than for them to make the call.*

***MSM_IDI_08***

## Packaging

*how sure are we that if I know my status, I will come to access care or I will call the hotline, we think a little, like a little counseling on HTS (HIV Testing Services) should be on the pack so that it can easily reduce the fear, it is not the end of the world, if you see it like this, it is like this, you will not die or yes there is drug for maintenance, all those little information that will really calm the nerve of whoever and all those like removing that clause or that veil of if you are HIV positive, you are dead or you are this, that the community has already paint those living with HIV with. If something like a little of such can be done inside the pack, with the hotline, it will be very very ok.*

***KOL FGD_01***

*A-Z: what I want you to do is that even though if this test kit is been approved, I think there should be a means, or something to show that this thing, we know our country, any new thing that is coming in, they normally fake it, they should make sure that if you want to get it, this is the sign that you will see that this thing is authentic or original, not that something that someone just seat down inside his house and create or redo it and it will look alike…*

***KOL FGD_02***

*I: Ok, so how would you want a test kit to look?*

*R7: May be smaller*

***MSM_IDI_07***

*I: But would you prefer it in another colour or it's just fine as white and blue?*

*R7: if it came in another colour, I would like it too...Right now, I'm just thinking yellow, pink*

***MSM_IDI_07***

*R8: for the kit, maybe they should have a special kind of bag for it*

***MSM_IDI_08***

*R8: Though the pack was okay, everything that was put inside the little bag, may be if the kit can have a better bag for it, may be they have a bag that house may be each section… Compartments*

***MSM_IDI_08***

*I feel it should be two kits in one not two separate kits...*

***MSM_IDI_17***

## Public awareness

*R5: ﻿Advertisement like it should be on TV like the channel...the news should carry it*

*﻿****MSM_IDI_05***

*TEWO: ... if we have you as a role model or as a face to the community or to the trained persons you have, you have a cohort of your clique, you know your persons, you know your friends and you serve as someone that you can even send may be particular text message or maybe you form a particular WhatsApp group with your friends, you pass out health tips on that platform, we have different channels which you can pass out information to them…*

***KOL FGD_01***

*DOUBLE D: if this kit is going to be sold, like I can collect it from the pharmacy and there is nobody, except it is being given to me by a KOL, then that KOL will be able to monitor me but once I get it from a pharmacy, is it the pharmacy attendant or the pharmacist that want to monitor me? That is one issue, so like during the first stage I said there should be audio and visual... advertise this kit audio and visually to the public, when I got to Alimosho General Hospital, I discovered that as we are doing training here, they are also training staffs there for this oral self-kit… the community were at the hospital to know more, everything we do here but their own was based on the general population, so for us to be able to get more of them if it's going to be going viral, there should be audio and visual...they should advertise it in radio, TV like social media.*

***KOL FGD_01***

*R 11: maybe they can try to let people know about it very well, so it will be more easier than going to the clinic to check their status, some people might not go, so it's good to let them know the self-test kit so that they can be coming down here… is like people that you are giving, so you can tell them, they should tell their friends, this and that... family and friends like that.*

***MSM_IDI_10***

*R 12: There are so many ways to pass it, through social media, TV, radio, can pass it through…*

***MSM_IDI_11***

*R17: It still boils down to educating people and sensitizing people, yes, like a couple of years ago if you spoke about HIV, I'll be like [Wears a scary look] Oh my God! Oh my God! Oh my God! But now if you say let's go for a testing I'm like ye! ride on, let's go, now it's not because I feel oh I'm the safest person or all of that, there are multiple ways to catch on to this thing, it's just sensitizing people, making people understand that look this thing there are ways you can go around it, you can still live a beautiful life if you stick to your meds and live a healthy life, eat well and all of those things.*

***MSM_IDI_17***

*﻿R17: ﻿Education of the younger generation because you see once the younger generation is sensitized it continues to ripple, because the older generation now were not properly sensitized, all we saw was once you say you have HIV you are dead! It's now that we are seeing that oh you can have it and you can live, I like the fact that the ARV drug are readily available, they don't have to pay for it, once you can start telling this young people hey! Don’t get it o, but if you do you can live, you can protect yourself, you can protect the next person and it is free, Nigerians like to hear the word, in fact people in general want to hear free! So once you can sensitize, go to schools. So you see it still boils down to educating the people.*

*﻿* ***MSM_IDI_17***

*R 20: Yes, there should be an advert, create more awareness about this, like on TV, and when you people are doing your advert it shouldn't be the normal skeleton they always show to scare people off about HIV, I think it should be much more different…*

***MSM_IDI_18***

## Referral

*JENNIFER: …if there's another way we can have other facilities we refer clients to, for instance, if you can't come to Pop Council, is not a must you come to Pop Council, if you are reactive, go to Alimosho or go to any near, general hospital if you don't want to come here, is not by force to come to this place, so if you can put this thing there. Let them know that there is different facility that they can go to, let's list it out there inside the test kit.*

***KOL FGD_01***

# USER PERCEPTION AND EXPERIENCES

This describes the perceptions and experiences of MSMs with the HIVST kit.

## Action following use of kit

### Communication with someone

*So what happened is that after getting the result, I called Peter, Peter Kass, I called him back*

***MSM_IDI_01***

*No, but I sent on WhatsApp, I sent "it was nice, thank you" that was what I text.*

***MSM_IDI_02***

*R6: I did not because for me, if I'm going to actually take that step with someone, it's going to be someone I know well and trust…*

***MSM_IDI_06***

*R8: Yes! Yes! something led to the topic, I think we were talking about technology or stuff and I talked about it, that people these days don't take out blood again that new oral self you know your HIV status*

***MSM_IDI_08***

*R 14: I just spoke with Ikenna, because Ikenna was the one who referred me to the study, I just felt it was not necessary to call the hot line*

***MSM_IDI_13***

*this time I waited for exact twenty minutes, then checked it, then it was single, then I called Miss Ebun immediately*

***MSM_IDI_14***

### Disposal issues

*I: How about disposition, how did you dispose of the kit after testing?*

*R1: The waste bin! I raised it up, I raised all the dirt up and I put it there*

***MSM_IDI_01***

*I: where did you dispose this self-test kit?*

*R2: I disposed it in the canal*

*I: You feel canal is more safe for you to dispose it*

*R2: No, for me am keeping it secret*

***MSM_IDI_02***

*R: I took it to the dust bin at home and just put it in the trash can*

***MSM_IDI_03***

*R6: I wrapped it up, I put it in a nylon, in the...nylon, make sure that the serum, you know the liquid thing didn't spill, tightened it up and threw away in the garbage.*

***MSM_IDI_06***

*R7: ...I put everything back into the bag and then I put it back into the white and blue bag, can't remember whether or not I put it in another nylon bag… even if a person saw it, may be like a roommate or something, I will just explain what it was, but I was just more concerned about probably if the chemical was not like something may be after some hours it may not be good for it to get to the skin... so I was more concerned about the health effect, the safety of the whole thing.*

***MSM_IDI_07***

*R8: I put it back into the pack and I disposed it in the bin*

***MSM_IDI_08***

*R: I think I cut it into pieces, then I tied it in a nylon and threw it away in the dust bin.*

*I: Do you think the way you cut it and the dust bin was safe for you?*

*R 14: Yes!*

***MSM_IDI_13***

*Well when I finished using the test and everything, I burnt it, I poured the liquid into the toilet, the water or chemical, and burnt everything then trashed it… If I had done so, Mmmm (he exclaimed) you know information spreads everywhere, throwing it to the waste bin just like that, I wouldn't be comfortable, because in my house I have little little kids there, so it will be like as if, no no!! I just decided tie it, burn it then dispose the ashes.*

***MSM_IDI_14***

*The disadvantages I don't know ooo, may be the disadvantage is in disposing it.*

***MSM_IDI_18***

*P.T 1: Yes! till now, I just made use of it then I wrapped it back in the pack. But I guess that's from my side, that’s my own problem. I just don't feel to dispose it yet. But I think if am to dispose it, I won't just dispose it anywhere. I will maybe put it in a bag, maybe a kind of black bag or something like that, just dispose it somewhere so no one will be able to read my result.*

***MSM_IDI_21***

### MSMs use of test kits

#### Abuse/Coercion

*R9: ...some people can decide to sell it, I guess, may be like you give me two kits now, I can decide to sell the second one for financial benefits*

***MSM_IDI_09***

*For example if my sex partner is asleep I can just (he laughed) go and swipe up and swipe down and just run the test on my own, those are means which the test can be abused.*

***MSM_IDI_13***

#### Friend

*I: How about other people, so if you get, would you bother sharing it with other people?*

*﻿R1: ﻿Yes, friends*

*﻿I: with friends, how about sexual partners?*

*﻿R1: ﻿Yes! Of course*

*﻿* ***MSM_IDI_01***

*R7: ...I am supposed to give it to someone cos my sister actually saw it and I told her about it and she said she wanted it, I said I will give it to her and then a friend of mine also wanted it so I was like, so people want it but I've actually not given it out not because I don't want to but I forgot to… for my sister I'm like... I'm not so sure, I would rather just may be want to give it to another guy*

***MSM_IDI_07***

#### Number of kits

*R 14: Two… Since there is an invalid result, the one would have been a waste, so I would recommend two*

***MSM_IDI_13***

*R2: For me you should give them one… if they see it they can buy it. If you give them two, they will use it and use it, they will not like buying again*

***MSM_IDI_02***

*R3: ...well, it shouldn't be...like two is okay because if you give the person one, it won't be enough because they may have someone they want to give and so at least minimum two, then maximum of the limit which you guys can go but I still think that two is enough, like minimum of two is actually nice,*

***MSM_IDI_03***

*I like the two test kits because if that is the case I think two test kits made sense why because you can easily fuck up the first one and go to the next one or you can even have invalid result on the first one and you can go to the next one, so you know that ok you've learnt from your mistakes and it wouldn't happen again, like for example if I wasn't smart enough while I poured the liquid away, the liquid has mistakenly poured away and I cannot retrieve it, that means that test kit is gone, that means I have to open the second test kit the same time but because I was able to manage that condition, that situation, so I was able to still use the same test kit.*

***MSM_IDI_15***

*M2: Well, I think people should be given more than one, let's say four*

*﻿* ***MSM_IDI_22***

#### Partner testing

*R: No, no, no not that, my...my own scenario is kind of different but why is that I don't have a stable partner that’s the problem, I only have sex when I feel like.*

*﻿I: ﻿Oh! Ok, so the partner testing for you is for people who have regular partners*

*﻿R1: ﻿regular partners!*

*﻿* ***MSM_IDI_01***

*I: How about people testing with their partners?*

*R3: It's a good initiative in the sense that at least you know what you are dealing with your partner and everything and if one person needs help, you can help the person as well.*

***MSM_IDI_03***

*R6: So yes, I can test, he can test, ok then, that's fantastic, now let's do partner testing, yes… I do like that! I do*

***MSM_IDI_06***

*R 11: The test kit is very ok, so it's nice, because i personally used on and I gave my partner to make use of it, so I like it very well*

***MSM_IDI_10***

*I: Do you think that there will be an issue among partners testing themselves or one testing an individual or one testing and the other one not testing, do you think there will be an issue?*

*R 15: Yeah!, because when someone hear about the word HIV, it saddens, what comes to your mind is that you ready to do it?, do you want to do it or something like that, and if there are two participant there, one wants to do it and the other one doesn't want to do it, it's going to cause an uprising*

***MSM_IDI_14***

*I: But for you, would you have been comfortable testing with a partner?*

*M2: Yea, if I feel there's a need for my partner to be there*

*I: What would be that need, what would prompt you to want to test with your partner?*

*R: If himself is testing his own or her own as well!*

***MSM_IDI_22***

#### Re-use after 1st kit

*R 2: The second one, I just used it like I used the first one, that’s what I did*

***MSM_IDI_02***

*R9: … I felt I could use it another time*

***MSM_IDI_09***

*I: The second test kit you told us you used it also, the same day?*

*R12: The same day*

***MSM_IDI_11***

*R 16: …the second kit, yes I did after 3 months…*

***MSM_IDI_15***

*R17: It's still there pending; I still gave myself the whole three months space to do it, by December I should check again.*

***MSM_IDI_17***

*P.T 1: ...the first kit I made use of, like I told you, that was the first time I was even getting tested, so I just have to use it to know my status. So, the second kit I was given, I made use of it within the three months because I got an infection, so I have to make use of it to know my status...*

***MSM_IDI_21***

*M2: Ah! I still have it o! I'm not giving it out to anybody, I will use it when the time comes, it's very fancy...*

***MSM_IDI_22***

#### Challenges

﻿*R4: ﻿Challenge that might arise is just that some other people might test positive and just want to keep it to themselves or something or some people might not really even have the mind to hold and may be they might just think suicide… I think that is the only challenge*

*﻿* ***MSM_IDI_04***

*R7: The disadvantages of the self-test kit, ...the time period, if someone extended it and then it didn't show the right result, I mean some people may not want to go ahead and may be the result is for example maybe it's meant to be positive and then just because there was a delay of something the person didn't do it well and then it gave the result that was not conclusive or it gave a result that looks like negative, so it's not healthy for the person, if it was me too like it's not healthy for me so that's the scary part that the person may just live in the fact that they don't know what's happening with them, they will be misinformed so that's the scary part…*

***MSM_IDI_07***

*R7: Of it, ok of people using it, I think the first thing is I don't, like everything that gets into the country that is meant to be free and may be for everybody, the fear is that people shouldn't hoard it and then start selling it or hoard it to divert it to another place…*

***MSM_IDI_07***

### Sharing of test results

#### Family members

*I: Okay, was he the only one you discussed your result with?*

*R9: Yea and my brother*

***MSM_IDI_09***

#### Friends

*I: Did you share your result with anybody?*

*R 16: Yes I have some two best friends, yes!!! Because we don’t keep anything from each other*

***MSM_IDI_15***

*I: did you share your result with anybody?*

*R 14: ... just my very close friend… I’ve heard rumors that he is affected also, so I think that was the only good part of sharing the result with him… He later confided in me that he too is, that is not the end and he gave me some questions to take…*

***MSM_IDI_13***

#### Helpline counselor

*R3: …I was given a call as well from Population Council in the sense that they asked if I did the test and what was the result and everything, which I shared as well.*

***MSM_IDI_03***

*R 18: No!!! I can't just go out and tell my neighbor I did something, but here when you guys call me for the second time, I told you what the result was*

***MSM_IDI_16***

*R11: they called me and I told them about my result...*

***MSM_IDI_10***

*R17: Yea, just to keep, just to keep them abreast of okay this is the kit you brought to me, I have used it and this is you know the result that I got from it.*

***MSM_IDI_17***

*P.T 1: It was the study line (hotline), they called me to know how I was able to make use of the kit and everything, As of that time I was kind of having a kind of slight problem, I kind of got an infection, so I have to like disclose my status to know what to do and to know better how to take care of myself.*

***MSM_IDI_21***

#### Partner

*I: Where you comfortable sharing your result with your partner, because you told us you shared your result with him?*

*R 13: Yes I share... we share everything, I was comfortable*

***MSM_IDI_12***

*I: why did you decide to share your result with that pharmacist?*

*R2: Because one: he is my personal friend, that's why and again he knows everything about Doctors, he treats my brothers like that, that's why I decided to have a contact with him… A sexual friend*

***MSM_IDI_02***

## Experience with the use of HIVST

### Reaction after use

#### Dislikes

*R6: I do not also like going to pharmacies to ask them for stuff, oh I want to do my STD test, I want to find out if I have AIDS cos they are very very judgy, I would have preferred for the answers, not like when you use the test, it brings out something like T and CT, I can't really remember but I would have preferred it to be a positive or negative, that would have made it so much easier cos I had to check to like o wait...what does this mean, you know*

***MSM_IDI_06***

*What I see as the disadvantage was the time, the timing which study is been carried, you know for them to give one person the kit, it takes too much time, the questions; which my clients were like, they were complaining that we came here so so time, look at the time we are leaving (they expressed displeasure) because of the question is too much, that's the disadvantage.*

***KOL FGD_02***

*...the eligibility criteria and filling forms for them and the interview which took a lot of time at the beginning phase. It was an issue between me and my client because most of them we have told them we would waste more than 30 minutes and you could stand here for more than 30, 40, one hour, two hours they have not attended to you, most of them were pissed off at a certain... they felt they are wasting their time at that moment in time which they had other things to do, which they created out that period of time because they felt the few minutes it's going to be that was told them that it didn't come out within that few minutes, it extended more than that time which we gave out to them, That was the main disadvantage*

***KOL FGD_02***

*I think disposal, if you are not in a safe zone or something, you get what I'm trying to say, if you don't have a good place for you to do this kind of thing and it's not something you can do anywhere*

***MSM_IDI_03***

*R5: I hate suspense*

***MSM_IDI_05***

*I was not so clear with the instruction with the negative and the positive, that's what I don't like.*

***MSM_IDI_13***

*May be the disadvantage is that there is no one there to tell you about it right, it's just you and the kit.*

***MSM_IDI_16***

*R19: For me it's okay, although may be the only issue I had when I was doing the testing was probably I don't know if it is general but the liquid in the buffer in mine was very little, I had to slant it*

*I: So you feel the liquid is not enough, the reagent is not enough*

*R19: Yes*

***MSM_IDI_19***

*M2: I think it took more time to read the result... twenty minutes… I think that was like the exhausting twenty minutes of my life… waiting for that twenty minutes and trying to read the result yea, it was really exhausting mentally and emotionally*

***MSM_IDI_22***

#### Emotional feelings

*R 11: ... I felt ashamed of myself, getting that kind of result, because I don't think I personally should have that kind of a result, at least I have no choice.*

***MSM_IDI_10***

*M2: Performing the test, I felt the need to do it, now after the test, I felt relieved that I actually did it, I felt this sort of relief because regardless of what the result would be, at least I know I would know my status, so after the test and I got to know my status, I even felt more relieved with my status.*

***MSM_IDI_22***

*R 14: No! I wasn't comfortable that's why I have to come for the confirmatory test*

***MSM_IDI_13***

*R 12: ... I felt down, I felt like I needed like a check. I learnt that there are two test kits there so that you can also recheck for confirmation, so I rechecked with the second one, it if was true and they gave me the same result, which was true...which was like ok. And then I have to calm down first and relax...and I will be fine. Basically, at the moment I was sad, I had to go out for a jug, I had to clean up myself and get ready…*

***MSM_IDI_11***

*R 13: I felt relieved, that was the idea of taking the test in the first place, so, I wanted to know my status there and then in a confidential way... I felt relieved that I was negative*

***MSM_IDI_12***

*R 15: Well, I wasn't the one that actually read the result, I gave it to him, take! Take!!! That was my result. He asked me several times, are you sure you want to see it? yes ... say it, say it… all my body was like cold, I couldn't do anything, I was just holding myself like this (he demonstrated), then he read it. For like three minutes I didn't look up (he laughed) was just like (he demonstrated) what happened, what happened, what’s going on, what's going on (he questioned), then he now said it's ok, comforted me… I was shocked about what I saw but at the same time, I was happy because I now know my status, so I know the next step to take…*

***MSM_IDI_14***

*…why would you want to destroy me while trying to help me, why are you telling me that am free for now, that means you found something that I cannot see, that you are not telling me about, am a very very sensitive person, I read a whole lot of meaning into the way we speak. So, that hunted me every day of my life till I did the next test. So, I have no body to tell me that kind of story, that was why I didn't call any helpline, I rather just read it myself and understand it the way I want to understand it than someone use a voice to say it in a way that is not the best way to say it to me.*

***MSM_IDI_15***

*M2: Wow! You have no idea, I felt very relieved… I felt quite relieved because before then, I have not actually done any HIV test, I think the last one I did it was like a year before then.*

***MSM_IDI_22***

#### Likes

##### **Confidentiality**

*It was really a nice experience from my side because every participant I referred from my side for the self-test really did well, they showed there support to participate for the self-testing because most of them felt it was the safest thing to do because it was something confidential, that which they know it was something between them alone, it's just for them to share their result if it was meant to be a complication later on*

***KOL FGD_02***

*The HIV self-test is very very impressive because I must commend that... it was quite very impressive because the few people I recruited, one of the person I recruited he was like ah! God how I wish if this is going to continue, for me to come back and get the test kit in three months time without anybody getting to know my result. It is very very confidential,*

***KOL FGD_02***

*the advantage is what I just said earlier, the  community they really really appreciated the opportunity for making them know there HIV status confidentially by their self alone and the follow up.*

***KOL FGD_02***

*﻿The advantage there is the confidential part of it, being alone yourself, knowing your status for yourself and do the judgement by yourself,*

***KOL FGD_02***

*R9: Yea, cos it was something I could use when I'm home alone without anybody intruding into my privacy so I felt it was good*

***MSM_IDI_09***

*The advantages of the test kit is gives you privacy, trust... you can trust on yourself and yourself alone*

***MSM_IDI_15***

##### **Convenience**

*It was a wonderful experience basically, we had in the community, should I say an issue we have is people not coming out to find out their HIV status or most especially coming into the facility because they think they are going to be stigmatized or discriminated or one thing or the other. So, this self-test kit made things so easier, being the fact that they could come and take this and go back to their own house and do their test themselves, read there result and all*

***KOL FGD_02***

*the self-kit is much more better than the regular way people do take because it's on your own convenience and your own space, your own time, when you are feeling willing to do it… get to be calm, you get to do it at your own leisure time*

***MSM_IDI_03***

*R9: ...the things I like, well, it was really convenient*

***MSM_IDI_09***

*R 12: Yes, i did use the test kit and it was easy and convenient for me to use,*

***MSM_IDI_11***

##### **Ease of use**

*The experience I had was that I liked the test kit, because it was very easy than going to the hospital to check yourself, that's it.*

***MSM_IDI_02***

*Me myself am scared of it because they feel there is actually pain which the self-test kit have actually very easy, it's just to swipe your gum and which is actually the best thing, anybody could do that as far as you follow the instruction which they felt it's a place for them to  be free*

***KOL FGD_02***

*The things I like it was; one: I like it because it was very easy and also the way you explained to me while using it, I was like thinking whether it was hard but fortunately, I got use to it and it was very easy*

***MSM_IDI_02***

##### **No needle prick**

*R: it has very good advantage, it's safer because people do get scared of needles and syringe, so this method is easy, safer and the instruction is there, you can't use it again for someone else.*

***MSM_IDI_08***

*The advantage was that all of them really accepted it, they accepted it actually because one, many of them never wanted the normal test kit because of many people are afraid of pin which is the lancet, which they are very afraid of.*

***KOL FGD_02***

*but it's very easy to use, instead of that one you go there, they will sting you, at times the second, third day you still feel the pain, that's the thing.*

***MSM_IDI_02***

*no need of going to the hospital to prick yourself, the normal HIV test…*

***MSM_IDI_09***

*P.T 1: I felt really really cool making use of it. Because, as I said earlier it was my first time, because I know the normal processed through pricking of finger nail, so I was glad I didn't go through that process the first time and I got to make use of it and got my result myself, so I was glad making use of it for the first time.*

***MSM_IDI_21***

### Reaction during use

#### Nervous

*At some point I was nervous…*

***MSM_IDI_04***

*R6: ...I generally don't like test, any kind of test, no matter what is it, I always have apprehension and anxiety for it, so my feelings were...oh! what am I going to do, my mind went to some very very dark places even before getting the results you know, so I was just like thinking of the worst possible outcome, even though it's not the worst but for me, I was thinking of the worst possible outcome for me and there was a lot of apprehension and anxiety but I don't think that was generally towards the test itself, it was just towards me as a person.*

***MSM_IDI_06***

*R9: …I was nervous like what will be the outcome of the result and I was surprised that it actually worked... I was just nervous about the outcome… All through, I was nervous till when I saw the result like okay, fine*

***MSM_IDI_09***

*R17: I tried not to sit down and just look at the reading for a whole twenty minutes, so I walked about still thinking o what is it like, at some point I glanced at it and I thought I saw two and my heart skipped a bit [Laughing] then I looked clearly and it was one and I was like...*

***MSM_IDI_17***

*I was just checking time, time was not even moving at all, it was very long, but when the test came out (ah!!! hived a sigh of relief)... normally whenever am anxious there is this cold sweat that comes out, I don't know why, it does just as my anxiety kicks in. So, when it did, the test came out, I was relieved to a point..*

***MSM_IDI_18***

*I: you said some of them had invalid result, Yes I think he responded. How did they feel when they had that result?*

*Gideon continued: They were scared, which I actually asked them to come here for their test. They were like oh! (they expressed surprise) will they do another one? And I was like yes! You have to do another one.*

***KOL FGD_02***

*I: Your friend, why actually did you call him to be there?*

*R 15: Because I was scared one, I needed someone; he is the kind of person who knows how to comfort someone. So, I needed someone to comfort me... so I called him immediately and he came immediately*

***MSM_IDI_14***

#### Relaxed/Calm

*R7: I won't say nervous cos like I said I gave it a few weeks before I used the kit but yea, the whole anticipation of doing something was there, it was just there, it was interesting to perform [Laughs]*

***MSM_IDI_07***

*R 10: I was rest assured, when I was using it, because due to the explanation given to me, I don't think it can do me any harm, I was free*

***MSM_IDI_20***

*M2:  No, I felt very good…*

***MSM_IDI_22***

## Instructions and guidelines

### Adherence to instructions

#### Expiring date

*I: Did you check on the expiring date?*

*R 15: No! I didn't check on the expiring date of the stuff, I just opened it and did it*

***MSM_IDI_14***

*I: Did you look towards the expiring date?*

*R 16: Yes I did… I didn't see it as too far (he stressed it) I cannot remember clearly what the expiring date of the one I used was because it's been a while but I knew that it wasn't too near, it wasn't too far because am a very sensitive person on that side. If it was I probably wouldn't have used it, but I looked at the expiring date.*

***MSM_IDI_15***

*I: Did you also check out for the expiring date?*

*R 18: Yes, I did… its ok, that's the first thing in Nigeria, I don't know anywhere else. You have to check about what you are using and anything else expiring date or the NAFDAC number*

***MSM_IDI_16***

*M2: Well, I think I’ve forgotten the expiration date but... I know I checked it that day and it hasn't expired as at then. Well, if it's anything within a year or eighteen months, it's ok for me*

***MSM_IDI_22***

#### Procedure

*Taking it, it's very simple! It's very very simple as in I just used it to touch the upper gum, then I brought it down to the lower then I inserted it inside the other stuff that has a little liquid water and I waited for the result for twenty minutes.*

***MSM_IDI_01***

*R8: ...at first when I wanted to use it, I thought it would be very difficult, I watched the video then I read the instructions and I just did as simple as that, so no one would find it difficult to use because it's very straight to the point. Taking the swab, it's very easy, just to the left and down to the right, it's very easy*

***MSM_IDI_08***

#### Timing

*R6: Twenty minutes, yea, there is a wait period and then I forgot everything and when I saw it I was like what does this mean! What does this mean! [Laughing]*

***MSM_IDI_06***

*R7: I think I waited, I used a stop watch...yea and then, the exact time, may be just some few minutes more and yea and I checked*

***MSM_IDI_07***

*I can't remember how long he told me, but me, I actually waited longer even when my test came out I didn't want to see it, I waited for additional ten minutes and I prayed a little prayer (he laughed)*

***MSM_IDI_18***

### Comprehensibility

*I: now do you think there are errors on the guide?*

*R 15: No!! Because on the guide most of the question I wanted to ask were there, so I was convinced that it's good.*

***MSM_IDI_14***

*Yes! that is the part, what happened in that aspect is that I think there is something confusing in explaining how you get the result, because the way it stated that there is a line, you will see a line and another two lines and the way, there is one is not showing too well like it's not well stated I want to get the result because first of all when I see the first one, my liver...as in...I felt so scared that what is this, I now go through it again, now find out that it was not what I was thinking about.*

***MSM_IDI_01***

*That language is that, during using it to know the side where it will get to and to know whether it is HIV or positive, that's where I was like confused. I was thinking maybe I should come back here, but when I asked someone that's a Doctor in my area, he said that I was like negative, I didn't have HIV…*

***MSM_IDI_02***

*R5: No, I think the information was enough*

***MSM_IDI_05***

*It was well balanced, there wasn't really a downside for me, the information was well and they gave me pamphlet, explained it to me, if anything was kind of way too much information cos, I'm not that slow, besides the positive, negative, nothing else, there were no errors, neither grammatical, it was concise, it was straight to the point, there were pictures depicting essentially each stage you know, so I would say, I would say it was very good, the information was quite clear.*

***MSM_IDI_06***

*R7: I think the kit was given out, it was okay, it was given out in an okay manner and then there was a manual in it that was self-explanatory and then there was also a video that was sent to us after the oral interview, so it was explanatory and once you've read the manual and you see the content, you would kind of like know what it's about…*

***MSM_IDI_07***

*R8: And it was as easy as I saw the video of how to use it and I tested negative with it… at first when I wanted to use it, I thought it would be very difficult, I watched the video then I read the instructions and I just did as simple as that, so no one would find it difficult to use because it's very straight to the point...*

***MSM_IDI_08***

*R9: It was actually very good, they tried their possible best to educate us about the kit, yea, all the information on how to use it was really helpful.*

***MSM_IDI_09***

*I think that there's enough information on the card in case your result comes out negative or positive... people you can reach for counseling and you know the next step you need to do, so I think, I think it's a win win basically.*

***MSM_IDI_17***

*P.T 1: To my own knowledge, the manner I received it was good, I was provided with enough information on how to make use of the kit and apart from that, it was my actual first time of getting tested, I haven't gotten tested before. It was actually my first time...actually this stuff is great and at least I got to test myself at my own convenient time and got to know my result before going through any other process..*

***MSM_IDI_21***

### Language preference

*I: So, how would you have preferred reading the result so that you can understand it?*

*R 2: My own is that the language should be negative (N) positive (P)*

***MSM_IDI_02***

*R 11: English is a general language, so English is Ok.*

***MSM_IDI_10***

*R 12: Maybe they can add like a local language, which will make it easier for some people to understand*

***MSM_IDI_11***

*I: What of the language, the English that we used, are you ok, do you think we need to translate it?*

*R 14: To other languages (he responded) no no no!!!, English is ok*

***MSM_IDI_13***

*R19: May be translating to other languages*

*I: Okay, which languages are you proposing?*

*R19: ...for example we are in Lagos, so may be Yoruba would have been good, may be pidgin English for those who can't understand proper English*

***MSM_IDI_19***

### Preferred instruction type

#### Pictorial

*Simpler in the sense that someone like a layman can also understand but that's the use of the diagram, the diagrams help, that's another way in which if you can't read very well, you can see the diagram to follow the procedure.*

***MSM_IDI_03***

#### Video

*R8: No! The video was just perfect instructions for the kit.*

***MSM_IDI_08***

*R: but then I also saw a video, that also assist me, maybe the video should be sent to assist them with it, so it will make it easier. Maybe it should be put on Youtube you get or send to the persons phone, the person can also use it as a preview if the person doesn't really get to understand the language or how to use it from the instruction guide…*

***MSM_IDI_11***

*To overcome such challenges the video should be provided during when it is distributed, then anyone who is distributing the kit should provide the video also because it is not only the literate people that will use it, a lay man will also make use of it. For a layman to make use of it, without video it will be very very difficult for him to do it, there are some people who can't read, a video will be beneficial to such people, to overcome that challenge.*

***MSM_IDI_21***

## Non-use of HIVST

*FGD2: You had a client that didn't use his test kit?*

*Ikenna: it wasn't mine personally but I know a KOL who had a client that didn't use his kit but gave it out to his friend or something*

*FGD 2: Why didn't he want to use the test kit*

*Ikenna: he had already... (stammering: he couldn’t explain why) but I know he didn’t use the test kit*

***KOL FGD_02***

*I: And you were offered two kits, I'd like to know what you did with the kits.*

*R5: I left it at my friends place because I didn't want to take it home, I haven't used them*

***MSM_IDI_05***

### Actions following non-use

*I: Where did you hide it?*

*R5: It's in my friend's place… I told him to keep it for me; I want to enjoy my life a little... [Smiling]*

***MSM_IDI_05***

### Reasons for non-use

*R5: Yes, I don't want to arise any suspicion at home, so I didn't take it home, but now that I'm back in school, I intend to use it… Sometimes I get frightened that the result could be positive… may be the fear to see the result made me*

*I: Oh, that's another reason*

*R5: Exactly… Honestly eh, I'm going to use the kit next month, I don't want to see it this month*

***MSM_IDI_05***

## Perception of HIVST

*I think I was kind of skeptical, I was like is the kit not trying to say one can find HIV now in the saliva…*

***MSM_IDI_19***

### Acceptability

*SEYI: Ok, I love the fact that the health sector, especially in the aspect of HIV/AIDS is growing every day, ok, for the fact that we are implementing this self-test kit. You don't need to book an appointment for HIV testing in any clinic, it's handy, you can take it anywhere you are going, you can do your test at any convenient time for yourself*

***KOL FGD_01***

*DOYIN: You tell them this is what this is, you are free to accept or decline, it's all up to you, we don't have to hold you for anything, you sign your consent form, ensure you read all of that so that you know what you are getting into and for those that decided not to, no problem, you get.*

*I: What was the rate of those that said yes and?*

*DOYIN: Most people said yes, or let me say most eligible people said yes because there was the eligibility clause, so most eligible people said yes and then most of those people wanted to come again for the endline and then you will see that most them are asking can we get more*

***KOL FGD_01***

*So, it was a beautiful experience, it was really accepted by most MSM because of the confidentiality sake, so it was a wonderful one.*

***KOL FGD_02***

*from my personally experience I will say 85% accepted it because even from the people I recruited, I can say out of like let's say 20 it's only like one that refused coming back, probably because he felt he wasn't comfortable with his result but the rest they all have great feedbacks*

***KOL FGD_02***

*For me throughout this study I did not see any barriers (reaffirms) but the most thing that I love is that most of the people I recruited, they accepted it and I follow up on them, they open up, which am so happy.*

***KOL FGD_02***

*R: that it will save me the stress of going to the hospital, that the test kit itself is appropriately correct, it's very correct.*

***MSM_IDI_13***

*I think the whole thing was ok, I will rate it 10/10 for me it was just perfect*

***MSM_IDI_16***

### HIVST packaging

*... what I liked about the packaging is that it's very practical, like I said earlier, it easy to access…*

***MSM_IDI_01***

*The thing I like about the pack is that binding it was ok for me, by me and  besides the packaging, I will even thanks to God for giving me that type of packaging, that’s a good packaging I even like it… Everything about the packaging is ok.*

***MSM_IDI_02***

*R7: I mean the packaging was quite big [Smiling]*

***MSM_IDI_07***

*It's very easy to open, it's sealed and covered properly, it looks attractive, will I say, it's properly packed.*

***MSM_IDI_13***

*R19: May be the bag, for me I feel it was may be a little childish somehow the drawings on it*

***MSM_IDI_19***

#### Colour

*R6: [Smiling] I would have wanted something more colourful, yes!*

***MSM_IDI_06***

*R7: The colour is fine, I think the white is good and it gives room for all the test to be very obvious, only if they want to make it may be colour friendly so that may be some people may tend to like it in other colours…*

***MSM_IDI_07***

*I love white... come to my house everywhere plus ceiling, curtain plus everything is white. It gives me sanity, so white is my thing*

***MSM_IDI_15***

#### Size

*...the size is actually big a bit because it's not something you can put in your pocket or something, it's something that you need to carry in the bag or a small cellophane or something but it's not, I don't think it's possible for them to reduce the size but if they can reduce it...*

***MSM_IDI_03***

*R5: I really like the kit because it was very portable, it's something you could do anywhere and you don't need to go to the hospital, get pricked by needles, take your blood sample and wait, come back next day, it was nice, I really like the kit.*

***MSM_IDI_05***

*R9: Perhaps the...test tube, the liquid inside, I think it's too small*

*I: Oh, it would have been more*

*R9: Like much bigger in size*

*I: The container or the liquid inside*

*R9: The container*

*I: It would have been bigger*

*R9: Yea*

***MSM_IDI_09***

*It wasn't too big but it could occupy a lesser space, but it's still ok for like a new product*

***MSM_IDI_15***

*R17: ...the packaging was good, I just feel it was too big for what was in it… So if it could be like the size of the content can be reduced, .that would be better because I think, I just think it's a waste of extra space, that's it.*

***MSM_IDI_17***

### Location for testing

#### Apartment

*I: where did you take the test?*

*R1: It was in my room actually but I locked myself up*

***MSM_IDI_01***

*…the testing was done in my room*

***MSM_IDI_03***

*I: In your room, was that comfortable for you or is there any place you would have preferred?*

*﻿R4: ﻿It was very comfortable, ﻿where would have been more comfortable than my room...*

***MSM_IDI_04***

*R7: Yea, so I had it done in my room, I was alone, there was no one with me and then the swabbing, at first I thought it was going to hurt but it was actually ok but may be it could be softer*

***MSM_IDI_07***

*R8: While using it, while testing, it was very easy, very easy, opened it, used it, I was doing it with my roommates because they were there with me*

***MSM_IDI_08***

#### Private place

*Eh...any place that you think that's private enough for you to do something like that is okay by me.*

***MSM_IDI_03***

## Potential harm and unintended consequences

### Abuse and coercion

*R7: I think the first thing is I don't like everything that gets into the country that is meant to be free and may be for everybody, the fear is that people shouldn't hoard it and then start selling it or hoard it to divert it to another place…*

***MSM_IDI_07***

*R 2: It can be abused, it can be very abuse, by keeping it anyhow and again they can dump it for not using it for years that's it. All I know it can be abused.*

***MSM_IDI_02***

*The only abuse I think is if people start using it regularly without giving them a time frame, because I believe that them checking themselves every three three months is okay,*

***MSM_IDI_03***

*R6: I don't think anyone could abuse the kit except if they are extremely, extremely, extremely stupid because it's one off, except if you drink the liquid which is written quite there clearly, do not ingest, so I do not think anybody can abuse it but again, like I said I can't speak for everybody, I can speak for most, most people would not abuse this.*

***MSM_IDI_06***

*When it comes to abuse of this self-test kit, like people selling it or something ...No!!!... I don't advise that.*

***MSM_IDI_17***

*Testing the child without his/her knowledge, yes!, it's an abuse also, because  you have to tell your child, even though you want to test your child, if the child is old enough you have to let him or her know, because if at that point you test your child and your child doesn't know about what you are doing to him or her and later on you come to tell the child that ok!, what I did for you now is HIV test and you tested positive, ok if just say negative, the child will be at first asking you questions; why will you do that without telling me  yes why, by the time that it will be becomes positive and you tell the child, there are some children that will run away from the house, and before you know it, it will be difficult to help that person and then the  fault lies on who, it will be your fault why you did not tell your child before you are doing it, because your child answer to what you want to do is ok!*

***MSM_IDI_17***

*R17: …I feel that in the wrong hands some people might force it on other people, I am speaking from the part where I would say oh I would like you to try this, if you are clear then…*

***MSM_IDI_17***

*R 20: it depends, ok take for instance if it was free because we like free things, we are never tired of give me, we always want give me, even when we have we still want to take and keep. People will want to collect and keep and be hoarding, some will want to hoard it*

***MSM_IDI_18***

*R 20: YES!!! I can have a partner, my partner can be sleeping I will just open there mouth and use it like that (he laughed) I can do that, I can do that (he repeated) seriously, me I will do it… but then again I wouldn't care about the price if I want to know my partner status, because I will still go and buy it, when my partner is sleeping I will use it (he laughed), I don't even care about the money, I will use it (he laughed excitedly)*

***MSM_IDI_18***

### False positives/negatives

*R7: The disadvantages of the self-test kit, the time period if someone forgot to, if someone extended it and then it didn't show the right result, I mean some people may not want to go ahead and may be the result is for example maybe it's supposed to be, meant to be positive and then just because there was a delay of something the person didn't do it well and then it was, it gave the result that was not conclusive or it gave a result that looks like negative, so it's not healthy for the person, if it was me too like it's not healthy for me so that's the scary part that the person may just live in the fact that they don't know what's happening with them, they will be misinformed so, yea, that's the scary part…*

***MSM_IDI_07***

### Incorrect use

*﻿let's say for this oral self-kit, like a child can't...because he knows that it's going to be painful but looking at this self-kit and if you open it you won't see anything harmful there, so I believe it should just be strictly kept for the adult, only for the adult use, so a child will end up drinking the liquid, I don't know if it will be harmful or not…*

***MSM_IDI_08***

*people will bastardize the whole process and misuse some of it*

***MSM_IDI_04***

### Risk of self-harm/Mental health

*R: …perhaps if the person turns out to be positive, the person can decide to commit suicide or something.*

***MSM_IDI_09***

*I: I want to know how you would have felt if your result was positive…Would you have felt, apart from crying, like harming yourself?*

*R 2: Yeah! I will feel like harming myself, that was why I said, that will be the end of me staying on earth, that's it.*

*I: So you would have killed yourself*

*R 2: Yes! yes! (he laughed)*

***MSM_IDI_02***
